# A method to analyze time expression profiles demonstrated in a database of chili pepper fruit development

**DOI:** 10.1038/s41598-021-92672-4

**Published:** 2021-06-23

**Authors:** Christian Escoto-Sandoval, Alan Flores-Díaz, M. Humberto Reyes-Valdés, Neftalí Ochoa-Alejo, Octavio Martínez

**Affiliations:** 1 Centro de Investigación y de Estudios Avanzados del Instituto Politécnico Nacional (Cinvestav), Unidad de Genómica Avanzada (Langebio), Irapuato, Guanajuato 36824 Mexico; 2 Department of Plant Breeding, Universidad Autónoma Agraria Antonio Narro, Saltillo, Coahuila 25315 Mexico; 3 Centro de Investigación y de Estudios Avanzados del Instituto Politécnico Nacional (Cinvestav), Departamento de Ingeniería Genética, Unidad Irapuato, Irapuato, Guanajuato 36824 Mexico

**Keywords:** Computational biology and bioinformatics, Genetics, Molecular biology, Plant sciences

## Abstract

RNA-Seq experiments allow genome-wide estimation of relative gene expression. Estimation of gene expression at different time points generates time expression profiles of phenomena of interest, as for example fruit development. However, such profiles can be complex to analyze and interpret. We developed a methodology that transforms original RNA-Seq data from time course experiments into standardized expression profiles, which can be easily interpreted and analyzed. To exemplify this methodology we used RNA-Seq data obtained from 12 accessions of chili pepper (*Capsicum annuum* L.) during fruit development. All relevant data, as well as functions to perform analyses and interpretations from this experiment, were gathered into a publicly available R package: “*Salsa*”. Here we explain the rational of the methodology and exemplify the use of the package to obtain valuable insights into the multidimensional time expression changes that occur during chili pepper fruit development. We hope that this tool will be of interest for researchers studying fruit development in chili pepper as well as in other angiosperms.

## Introduction

Measurements of gene expression constitute the primary molecular phenotype. RNA-Seq experiments^1^ allow genome-wide estimation of the relative level of gene expression in a particular species, organ, tissue or even single cells^2^.

Temporal gene expression profiles consist in measurements of gene expression at consecutive times^3^, and from such data it is possible to estimate the transcriptome changes that occur during the progression of organ developing programs. Phenomena as seed development^4^, senescence^5^ and aging^6^ have been shown to be conserved in plants. In particular, the development of fleshy fruits—an indispensable part of the human diet, is probably conserved throughout the angiosperms^7^.

There is a plethora of software tools developed to analyze different aspects of RNA-Seq data^8,9^, many of them designed to perform differential gene expression. For example, the NCBI provides “GEO2R” (https://www.ncbi.nlm.nih.gov/geo/geo2r/), a tool to compare two or more groups of samples in order to identify genes that are differentially expressed across experimental conditions^10^. However, given that time expression profiles are multidimensional—generally with more than 3 consecutive times sampled, traditional statistical methods are of limited relevance, and new approaches are required^3^.

As mentioned above, the main challenge for the analysis and interpretation of time course experiments is their multidimensionality^11^. Assume that gene expression is measured at times $t_1, t_2, \ldots , t_n;\ (t_i < t_{i+1})$, and for each gene we have a vector of estimated expressions, say, ${\mathbf {g}} = (g_1, g_2, \ldots , g_n)$. Clearly, for all $n>2$, there is not a single univariate test that could classify complex expression patterns throughout time; in fact, we must perform at least $n - 1$ hypothesis tests on each pair of neighbor intervals, say, ${\mathcal {H}}_0 (1): g_{1} = g_{2};\ {\mathcal {H}}_0 (2): g_{2} = g_{3};\ \ldots ; {\mathcal {H}}_0 (n-1): g_{n-1} = g_{n}$, to be able to classify all possible time course patterns. Multidimensionality of time course experiments implies multi-testing, and this in turn opens the possibility of an “Error Type III”^12,13^, which consist on estimating the wrong ordering in a set of means.

One aim of the analysis of time course experiments could be to detect significant periodic modes in time, and examples of tools for this propose are the ones documented in^14^ and^15^. Other approach, closer to our interest here, is the identification of expression patterns in time course experiments. In^16^, the authors present an application combining modeling and a dimension reduction technique based in the ANOVA of simultaneous component analysis useful for microarray data. Also^17^ presents a test for microarray data that makes explicit use of the temporal order by fitting polynomial functions to temporal profiles. Other references for time course microarray experiments are cited in^3^, which also presents and discusses clustering and inference of networks from time course datasets.

With regard to the analysis of RNA-Seq time course experiments^18^, presents a linear mixed model spline framework. The proposed framework consists basically of three stages: (1) Identify and removal of “non-informative” profiles, (2) Modeling, *via* a serial model fitting approach to obtain smoothed profiles and (3) Analysis to identify similarities between summarized profiles by clustering, or hypothesis testing to identify differences over time. In this approach filtering “non-informative” profiles let out of the analysis genes that are relatively constant through the time frame explored, while fitting successive models of increasing complexity has the risk of ignoring patterns that are too convoluted to be fit with this spline approach.

A method to identify differential expression profiles in time course microarray experiments—implemented in the package “*maSigPro*”^19^, was updated to be able to use RNA-Seq time series analysis by introducing Generalized Linear Models (GLM) under the Negative Binomial distribution^20^. However, as its original version, the updated version of *maSigPro* relies on polynomials to fit time expression data through time. While the use of polynomials gives good results for cases when there are one or few critical points, either maxima or minima, it is well known that polynomial fitting fails when the behavior of the target function is too complex^21^. Because there is no guarantee that gene expression through time will always be simple, it appears better to look for a methodology that could fit even the most complex patterns shown by the data.

Chili pepper (*Capsicum* spp.) is an important crop, as well as a model for fruit development studies and domestication^22^. We developed a methodology to examine time expression profiles by testing neighboring time intervals and then obtaining “Standardized Expression Profiles” (SEPs), which can be easily interpreted and tested. That procedure was applied to all genes estimated in 12 accessions of chili pepper expressed during 7 temporal stages of fruit development. All curated data and functions to analyze them are included in the R^23^ package “Salsa”^24^. This data mining tool arouse during the course of a previous project, and has proven to be useful to reveal novel insights about the domestication of chili pepper^22^; please see https://www.mdpi.com/2223-7747/10/3/585. Here we explain the rationale of the methodology, present a panorama of its possibilities and exemplify the use of this tool.

## Materials and methods

### Biological materials and sequencing

The data in Salsa were obtained from a time course experiment estimating gene expression at seven time points of fruit development: 0, 10, 20, 30, 40, 50 and 60 days after anthesis (DAA), in 12 accessions of chili pepper. Table 1 presents the content of the data frame “acc” within the Salsa package.

**Table 1:** Information in the data frame “acc” within the R package Salsa.

acc.key	acc.type	acc.name
AS	D	Ancho San Luis
CM	D	Criollo de Morelos 334 (CM334)
CW	D	California Wonder
JE	D	Jalapeno Espinalteco
ST	D	Serrano Tampiqueno 74
ZU	D	Zunla-1
CO	W	Piquín Coahuila
QU	W	Piquín Queretaro
SR	W	Piquín Sonora Red
SY	W	Piquín Sonora Yellow
CQ	C	$F_1$: CM female $\times$ QU male
QC	C	$F_1$: QU female $\times$ CM male

Head of table in mono spaced font gives the names of the variables (acc.key, acc.type and acc.name), while cell content present possible values for those variables.

The content of data frame “acc”, displayed in Table 1, presents the accessions available in Salsa and helps in the use of the database. Variable “acc.key” contains a two letter code used within the package to distinguish each one of the 12 accessions studied. The categorical variable “acc.type” denotes if the accession is Domesticated (“D”), Wild (“W”) or a Cross (“C”) between a domesticated and a wild accession. Finally, variable “acc.name” gives a short description of the accession, corresponding with the common name used to denote the accession in the literature.

The RNA-Seq experiment from which Salsa data originated consisted in sampling fruits of the 12 accessions shown in Table 1 at 7 different times of development; this makes a total of $12 \times 7 = 84$ sampling points. For each combination of accession $\times$ time of development, 2 RNA-Seq libraries (biological replicates) were constructed, thus a total of $84 \times 2 = 168$ RNA-Seq libraries were employed to estimate time expression profiles. Additionally, some of the accessions were sampled in times larger than 60 DAA, but those data were not used for time profile estimation; please see Supplementary material in^22^. In total we obtained data from 179 RNA-Seq libraries, that comprise the 168 used for time profiles estimation plus some extra samples from times larger than 60 DAA as well as libraries from plantlet stage for one domesticated and one wild accession. Descriptions of these 179 libraries are in data frame “library.desc”, while raw counts of map reads for all genes at each one of the libraries is in data frame “readcounts”, both within the Salsa package. Sequencing, filtering and mapping of the raw reads to the reference *Capsicum* genome are presented in the supplementary methods and results in^22^. In total more that 3 billions of raw reads where map to the reference genome, and these data have been deposited in NCBI’s gene expression omnibus (GEO)^25^, and are accessible through GEO Series accession number GSE165448 (https://www.ncbi.nlm.nih.gov/geo/query/acc.cgi?acc=GSE165448).

### Estimation of standardized expression profiles (SEPs)

Previously, in^26^, we presented a methodology to classify time expression profiles into discrete classes, and here we extend such procedure to obtain “*standardized expression profiles*” (SEPs) in the general framework of an RNA-Seq time course experiment.

We will assume to have data from replicated RNA-Seq libraries at times $t_1, t_2, \ldots , t_n;\ (t_i < t_{i+1})$ (we must have at least two biological replicates for each time). We will also consider that the RNA-Seq libraries estimated a total of *g* genes for each one of the times. Now, consider only the $n-1$ contrasts taking two neighboring intervals, i.e., contrasts between times 1 and 2, 2 and 3, $\ldots$, $n-1$ and *n*. Those contrasts can be tested, for example by using the package “EdgeR”^27^, to obtain the *p*-values corresponding to the *g* genes at each one of the $n-1$ contrasts between neighboring intervals. For each gene at each contrast the null hypothesis of interest is ${\mathcal {H}}_0:\ \mu _{ij} = \mu _{i\ j+1}$, where $\mu _{ij},\ \mu _{i\ j+1}$ are the true means of expression for gene *i* at time *j*. If the null hypothesis is not rejected, then we will consider that the gene *i* is in a steady state, “S”, between times *j* and $j+1$. On the contrary, if the null hypothesis is rejected we have two possibilities, depending on the estimated values of expression, ${\hat{\mu }}_{ij}$ and ${\hat{\mu }}_{i\ j+1}$, say: the gene increased its expression if ${\hat{\mu }}_{ij} < {\hat{\mu }}_{i\ j+1}$, that we will denote by “I”, or, alternatively the gene decreased its expression if ${\hat{\mu }}_{ij} > {\hat{\mu }}_{i\ j+1}$, that we will denote by “D”. As result, the time expression profile of a gene can be summarized by a vector of $n-1$ symbols, where each symbol can be one of three alternatives, say, “S”, “I” of “D”. In symbols, the model for a gene *i* can be summarized as ${\mathbf {m}}_i = (m_{i1}, m_{i2}, \ldots , m_{i\ n-1})$ where $m_{ij} \in \{ \hbox {S, I, D}\}$. We call these models “Ternary Models”, because each one of the $t-1$ symbols can be one of three possibilities (S, I or D). Nonetheless, given that we are going to be performing thousands of tests, *p*-values need to be corrected by multi-testing, transforming them into *q*-values to obtain an acceptable “false discovery rate” (FDR)^28^.

In summary, the procedure described above gives a function that transforms the *t*-dimensional semi-continuous space of gene expression into a unidimensional space of $3^{\ t-1}$ discrete elements, constituted by all possible ${\mathbf {m}}_i$ models. This is so because the information for each gene from all RNA-Seq libraries is now summarized in symbolic vectors having three possibilities for each element, thus we have $3 \times 3 \times \cdots \times 3$ (t-1 times) possibilities for all possible models ${\mathbf {m}}_i$, and by performing such transformation we have used all relevant information about the expression of the gene in all *t* times, including the random error (unexplained variation) represented by differences between the replicates for each time, given by distinct RNA-Seq libraries. To give a numerical representation of a model ${\mathbf {m}}_i$ we first consider the mean estimates of expression at each one of the *t* times, say, the vector $\mathbf {{\hat{\mu }}}_i = ({\hat{\mu }}_{i1}, {\hat{\mu }}_{i2}, \ldots , {\hat{\mu }}_{it})$, where each estimated mean of expressed value, ${\hat{\mu }}_{ij}$, is the mean expression measured as fragments per kilobase of gene model per million mapped reads (FPKM)^29,30^. Finally, we calculate the SEP for a gene *i* as a vector ${\mathbf {s}}_i = (s_{i1}, s_{i2}, \ldots , s_{it})$, where $\sum _j s_{ij}=0$ and $S({\mathbf {s}}_i) = 1$, i.e., we use the pair $({\mathbf {m}}_i,\ \mathbf {{\hat{\mu }}}_i)$ to calculate an standardized version of the gene expression pattern which has mean of 0 and standard deviation, *S*(), equal to 1. For details of this procedure please see the “Supplementary Material” in^22^ as well as Additional file 1.

For the case of the chili pepper fruit data gathered in Salsa, we have 2 biological replicates of each RNA-Seq library for each combination of accession $\times$ time of fruit development. We have $t=7$ times of fruit development ($0, 10, \ldots , 60$ DAA), thus we have $7-1=6$ neighboring time intervals, and in this case the number of different ternary models (${\mathbf {m}}_i$) is $3^6=729$. The exactTest function of the R package “edgeR” (version 3.20.9) was used to obtain the *p*-values of the contrasts between the 6 neighboring time intervals for all 35883 genes annotated in the *Capsicum* reference genome in each one of the 12 accessions. Of the total of 35883 genes annotated, only 29946 ($\approx 83.45\%$) were consistently expressed in all the 12 accessions and thus only those genes were taken into account for SEPs estimation. An approximate 1% FDR was calculated for comparisons between two SEPs, and the edgeR results were used to construct the sets of ${\mathbf {m}}_i$ models for each gene and accession. Then mean expressions in FPKM units (data.frame “FPKM.expr” in Salsa) were used to obtain the SEP estimates for genes of the consistent set that were expressed in each accession (see Additional file 1 for details). This produced a total of 313919 estimated SEPs, allocated into the data.frame “SEP” in Salsa. This data frame contains—apart from the SEP estimate, identifiers for gene, accession, accession type as well as the ternary model (${\mathbf {m}}_i$) and the time at which the maximum standardized expression was reached.

**Figure 1:**
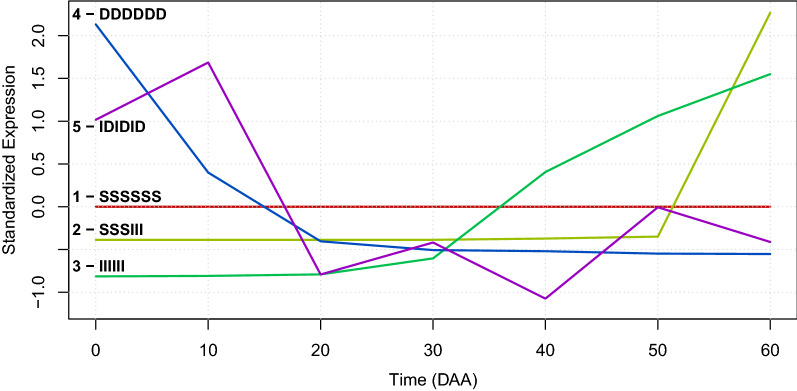
Examples of SEPs with different models (${\mathbf {m}}_i$) for 5 genes in accession “CM”. The number of case and symbolic representation of the model are shown in the legend at the left hand side of the plot for each one of the numeric representations of the SEPs (colored lines; see Table 2 for the description of the protein coded by each gene).

All possible $3^6=729$ different ternary models (${\mathbf {m}}_i$) appeared in the chili data, at different frequencies. Over all genes and accessions, the most frequent models were “DSSSSS”, “SSSSSS” and “ISSSSS” with frequencies of approximately 4, 3 and 2%, respectively. All other 726 models appeared at frequencies $\le 1\%$. Model “DSSSSS”, in $\approx 4\%$ of the cases, implies that gene expression decreased from the initial state at 0 DAA—the mature flower, to the next state as 10 DAA (this is shown as the initial “D” in the model), and then the gene stayed at an steady state (“S”) up to the end of the fruit development at 60 DAA. Genes with model “SSSSSS”, in $\approx 3\%$ of the cases, implies that gene expression remained in an steady state, “S”, during all the times intervals, while model “ISSSSS”, in $\approx 2\%$ of the cases, implies that the genes were at low expression in the mature flower, but increased from 0 to 10 DAA to then remain in a steady state up to the end of fruit development. Figure 1 present 5 examples of SEPs for genes with different models, while Table 2 gives the identifiers, models, and coded protein for each one of the cases presented in Fig. 1.

**Table 2:** Examples of 5 different ternary models (${\mathbf {m}}_i$) for genes expressed in accession “CM”.

Gene id	Model (${\mathbf {m}}_i$)	Protein coded by the gene
192	1—SSSSSS	tRNA-splicing endonuclease subunit Sen2-1-like
7969	2—SSSIII	Non-specific lipid-transfer protein A-like
1625	3—IIIIII	Uncharacterized GPI-anchored protein At3g06035-like
4076	4—DDDDDD	1-aminocyclopropane-1-carboxylate oxidase
27681	5—IDIDID	Probable calcium-binding protein CML36

In Fig. 1 we see graphic examples of SEPs with 5 different models (${\mathbf {m}}_i$). This shows that the original 7-dimensional space of expression profiles during fruit development can be transformed to a discrete space with only 729 models—of which Fig. 1 presents only 5 cases, and that the numerical representation of the time expression profiles as SEPs can give a graphic representation that is easy to interpret and contains the information necessary to group and contrast different time expression profiles.

### Testing differences between two SEP sets

SEPs are based on ternary models and summarize time expression profiles in scale free measurements. Thus, plotting the SEP of a gene the researcher immediately obtains an statistical summary of its behavior during the time course experiment. Additionally, SEPs can be group by criteria which include, for example, the origin (accession), or the Gene Ontology category (molecular function, biological process or cell component), or any other known attribute of the corresponding genes. On the other hand, the researcher could have sets of genes which are of interest from results obtained in previous studies, or in a different specie, etc.

Here we consider the problem of determining if two sets of SEPs say, ${\mathbf {S}}_A = \{{\mathbf {s}}_{A1}, {\mathbf {s}}_{A2}, \ldots , {\mathbf {s}}_{Ak}\}$ and ${\mathbf {S}}_B= \{{\mathbf {s}}_{B1}, {\mathbf {s}}_{B2}, \ldots , {\mathbf {s}}_{Br}\}$ are statistically equivalent, i.e., if they present the same or different average time profile, or in other words if they can or cannot be considered as having the same mean expression behavior over time. We will not impose any restriction on the nature of these two SEPs sets, and will ask only that the numbers of SEPs included at each one of them, say the numbers *k* and *r*, must be both larger than two ($k> 2;\ r > 2$), but they could be different ($k \ne r$).

To test the difference between ${\mathbf {S}}_A$ and ${\mathbf {S}}_B$ we propose to use the Euclidean distances between and within the components of the two groups. For arbitrary vectors of dimension *n*, say ${\mathbf {x}} = (x_1, x_2, \ldots , x_n);\ {\mathbf {y}} = (y_1, y_2, \ldots , y_n)$, their Euclidean distance is defined as $d({\mathbf {x}}, {\mathbf {y}}) = \sqrt{\sum (x_i - y_i)^2}$. Now consider the vector formed by the Euclidean distances *between* all different pairs of SEPs, where the first SEP belongs to ${\mathbf {S}}_A$ and the second to ${\mathbf {S}}_B$, say ${\mathbf {D}}_B = (d({\mathbf {s}}_{A1}, {\mathbf {s}}_{B1}), d({\mathbf {s}}_{A1}, {\mathbf {s}}_{B2}), \ldots , d({\mathbf {s}}_{Ak}, {\mathbf {s}}_{Br}))$. The vector of Euclidean distances between SEPs in ${\mathbf {S}}_A$ and ${\mathbf {S}}_B$ has dimension $k \times r$. Now we will construct the vector of Euclidean distances *within* different SEPs in the two groups, say ${\mathbf {D}}_W = (d({\mathbf {s}}_{A1}, {\mathbf {s}}_{A2}), d({\mathbf {s}}_{A1}, {\mathbf {s}}_{A3}), \ldots , d({\mathbf {s}}_{A k-1}, {\mathbf {s}}_{Ak}), d({\mathbf {s}}_{B1}, {\mathbf {s}}_{B2}), d({\mathbf {s}}_{B1}, {\mathbf {s}}_{B3}), \ldots , d({\mathbf {s}}_{B r-1}, {\mathbf {s}}_{Br}))$; in words, to obtain ${\mathbf {D}}_W$ we take in turn all possible different pairs of SEPs within ${\mathbf {S}}_A$ and also within ${\mathbf {S}}_B$, thus the dimension of this vector is $k(k-1)/2 + r(r-1)/2$.

The biological hypothesis that the two SEP sets have statistically equivalent time profiles can be translated to the null hypothesis ${\mathcal {H}}_0:\ \bar{{\mathbf {D}}}_B = \bar{{\mathbf {D}}}_W$ that need to be contrasted with the one-tail alternative hypothesis ${\mathcal {H}}_a:\ \bar{{\mathbf {D}}}_B > \bar{{\mathbf {D}}}_W$ (here $\bar{{\mathbf {D}}}_B$ and $\bar{{\mathbf {D}}}_W$ are the *true* means of the distances between and within SEPs, respectively). The rational to suggest this test is that the vector of estimated distances between members of the two sets, ${{\mathbf {D}}}_B$, measures how far apart are members of the two groups in the *t*-dimensional space, while the distances within the two groups, estimated by ${{\mathbf {D}}}_B$, measure the heterogeneity or “noise” that exist within the SEPs in each one of the two groups. Thus we will consider that if the null hypothesis ${\mathcal {H}}_0:\ \bar{{\mathbf {D}}}_B = \bar{{\mathbf {D}}}_W$ is rejected in favor of the one tail alternative, ${\mathcal {H}}_a:\ \bar{{\mathbf {D}}}_B > \bar{{\mathbf {D}}}_W$, then we can consider that the two sets of SEPs represent different mean time expression profiles. This rational is parallel to the one employed in classical ANOVA, where the variation *within* treatments is used as a measure of noise (unexplained error), while the variation *between* treatments is the one of interest.

Now, we must select a statistical test to decide between ${\mathcal {H}}_0:\ \bar{{\mathbf {D}}}_B = \bar{{\mathbf {D}}}_W$ against ${\mathcal {H}}_a:\ \bar{{\mathbf {D}}}_B > \bar{{\mathbf {D}}}_W$. To take this decision it is illustrative to consider how the number of elements in the vectors ${\mathbf {D}}_B$ and ${\mathbf {D}}_W$ (from which the mean distances $\bar{{\mathbf {D}}}_B$ and $\bar{{\mathbf {D}}}_W$ are estimated) grow as function of the number of elements in ${\mathbf {S}}_A$ and ${\mathbf {S}}_B$. We have mentioned before the lengths of the vectors: $|{\mathbf {S}}_A| = k$, $|{\mathbf {S}}_B| = r$, $|{\mathbf {D}}_B| = k \times r$ and $|{\mathbf {D}}_W| = k(k-1)/2 + r(r-1)/2$. Table 3 presents examples of the lengths of the vectors $|{\mathbf {D}}_B|$ and $|{\mathbf {D}}_W|$ for some values of *k* and *r*.

**Table 3:** Lengths of the vectors ${\mathbf {D}}_B$ and ${\mathbf {D}}_W$ ($|{\mathbf {D}}_B|$ and $|{\mathbf {D}}_W|$, respectively) for some values of *k* and *r*.

*k*	*r*	$|{\mathbf {D}}_B|$	$|{\mathbf {D}}_W|$	Total: $|{\mathbf {D}}_B| + |{\mathbf {D}}_W|$
2	2	4	2	6
2	5	10	11	21
2	10	20	46	66
5	2	10	11	21
5	5	25	20	45
5	10	50	55	105
10	2	20	46	66
10	5	50	55	105
10	10	100	90	190
$\cdots$	$\cdots$	$\cdots$	$\cdots$	$\cdots$
15	15	225	210	435

In Table 3 we can see how the number of values taken into account to estimate the mean distances between and within SEPs elements ($\bar{{\mathbf {D}}}_B$ and $\bar{{\mathbf {D}}}_W$) rapidly grows in a multiplicative fashion with the increase in the numbers of elements in the original SEP sets, ${\mathbf {S}}_A$ (of size *k*) and ${\mathbf {S}}_B$ (of size *r*). For example, for values of $k \ge 5,\ r \ge 5$ the number of distances used to estimate $\bar{{\mathbf {D}}}_B$ and $\bar{{\mathbf {D}}}_W$ are $\ge 20$, thus the distributions of the estimated mean distances will be approximately normal, leading to the selection of the one tail Student’s *t*-test to decide between ${\mathcal {H}}_0:\ \bar{{\mathbf {D}}}_B = \bar{{\mathbf {D}}}_W$ against ${\mathcal {H}}_a:\ \bar{{\mathbf {D}}}_B > \bar{{\mathbf {D}}}_W$. The selection of this particular hypothesis test is supported by the results presented in^31^. Additionally, the vectors ${\mathbf {D}}_B$ and ${\mathbf {D}}_W$ have approximately independent distributions, and results of the Shapiro-Wilk normality test applied to the residuals ${\mathbf {D}}_B - \bar{{\mathbf {D}}}_B$ and ${\mathbf {D}}_W - \bar{{\mathbf {D}}}_W$ do not show strong normality departures (results not shown). Also, applying the non parametric Wilcoxon test give *p*-values that are highly concordant with the ones obtained by the *t*-test in all cases assayed (results not shown).

In summary, we propose that the application of the *t*-test on the distances between and within SEPs elements is useful to decide if two sets of SEPs are equal in average. Salsa function “analyze.2.SEPs” implements this procedure for the chili pepper data.

### Structure, data and functions in Salsa

Salsa is organized as a relational database^32^. This means that different data aspects are organized into data frames which are connected among them by common variables. Table 4 presents the main data frames and functions that constitute Salsa.

**Table 4:** Main data frames (rows “d.”) and functions (rows “f.”) in Salsa.

Row	Name	Short description
d.1	gene	Gene description
d.2	readcounts	Read counts for each gene at each library
d.3	FPKM.expr	Expression data in FPKM
d.4	SEP	Standardized expression profiles (SEPs)
d.5	SEP.id	Summaries of SEPs for each gene expressed
d.6	GO.annot	GO annotations
d.7	all.GO	Gene ontology (GO) annotations
d.8	acc	Accessions
d.9	library.desc	Description of RNA-Seq libraries
f.1	gene.summary	Graphic and numeric summary of a gene
f.2	get.ids	Selects a set of gene identifiers
f.3	get.SEP	Obtains a SEP data frame from various criteria
f.4	SEPs.plot	Plot mean expression times in SEPs
f.5	SEP.summary	Summary of a SEP dataframe
f.6	analyze.2.SEPs	Test two SEPs trough Euclidean distances
f.7	analyze.GO	GO enrichment analysis (single term)

In Table 4 data frames in rows d.1 to d.6 include the variable “id”, a unique numerical gene identifier which links different data attributes for each gene, as its description (d.1), expression values (d.2 and d.3), estimated SEP and SEP summary (d.4 and d.5, respectively) and available GO annotations (d.6). On the other hand, data frames d.7, d.8 and d.9 contain all GO aspects, accessions and libraries, respectively.

**Figure 2:**
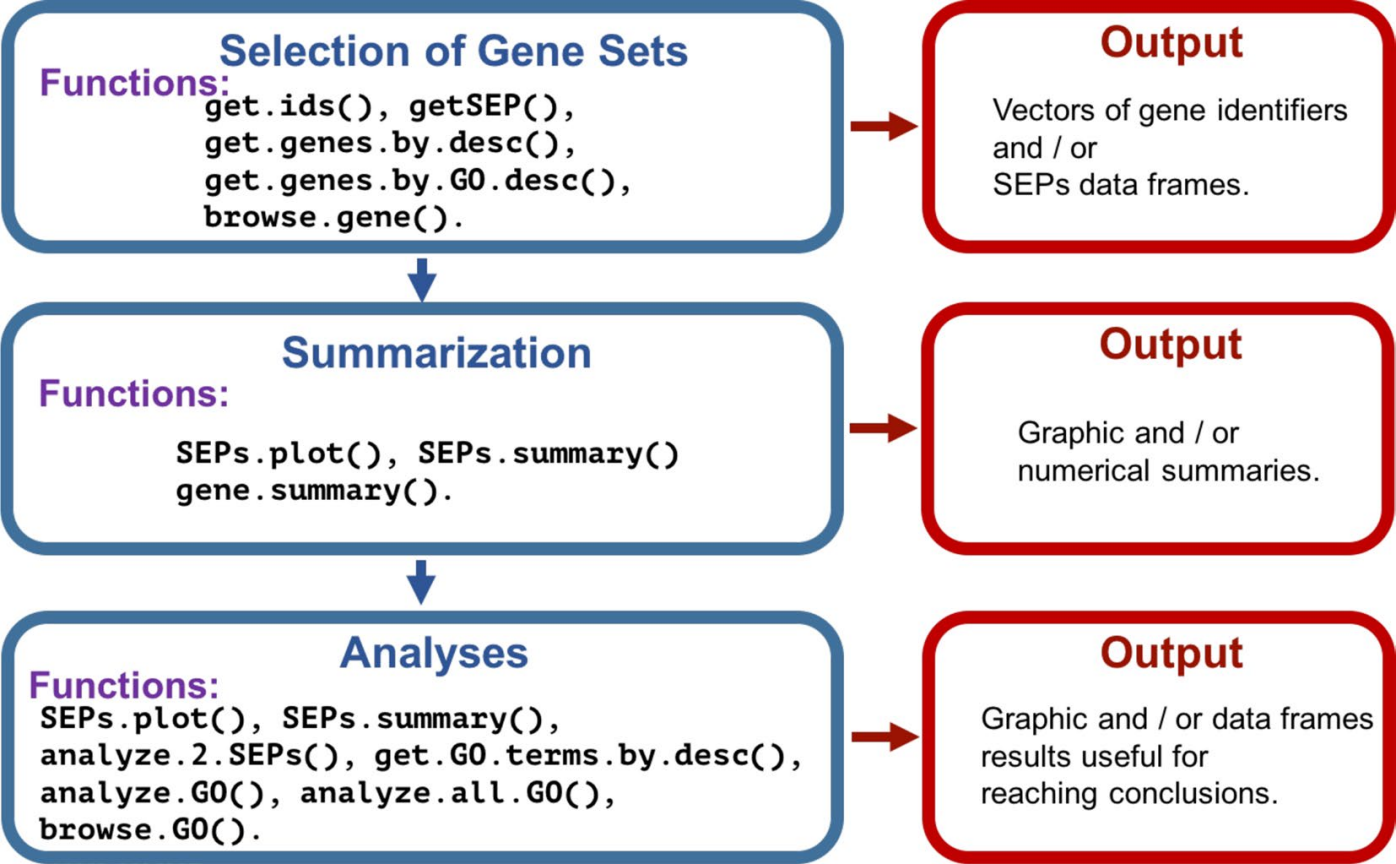
Simplified schema of data mining workflow in Salsa. Comprehensive description of the output of each one of the functions, as well as details for their use are presented in Additional file 1 as well as in the Salsa package manual.

Main functions in Salsa are presented in rows f.1 to f.7 in Table 4. The gene.summary function (f.1) plots the SEPs of a gene grouped by type (D, W and C) and outputs an statistical summary. Function get.ids (f.2) allows the selection of gene identifiers that share a description or other attributes. The get.SEP function (f.3) is the core of the Salsa querying algorithm, because it allows to obtain a set of SEPs fulfilling different criteria, as gene identifiers or description, accession key or type and model, among others. Functions in rows f.4, f.5 and f.6 plot, summarize and test one or more SEPs data frames, respectively. Finally, function analyze.GO in f.7 perform a GO enrichment analysis for a target set of genes in a given GO aspect. For brevity, other complementary data and functions are not shown in Table 4, but they are documented in the manual of the package as well as in Additional file 1. Figure 2 presents a simplified schema of data mining workflow in Salsa.

## Results and discussion

### Data mining possibilities with Salsa

Data mining, also called “knowledge discovery”, is the process of uncovering interesting and useful patterns and relationships in large volumes of data (https://www.britannica.com/technology/data-mining“Data mining” entry at Britannica). In the context of gene expression, data mining has been used for example to obtain^33,34^ and visualize networks^35^, or to find association rules^36^.

Figure 2 presents a simplified schema of data mining workflow in Salsa.

The first step to initiate an analysis in Salsa is to select one or more sets of *interesting* genes (upper rectangles in Fig. 2). This is the most difficult phase to explain, because it depends on the specific and limitless interests of researchers. As shown in the figure, various functions can help with the delimitation of such sets. The most important and flexible of these functions is “get.SEP()”. This function has 10 parameters to select specific gene attributes. These parameters are “ids”, “descr”, “acc.key”, “acc.type”, “model”, “ExistInAll”, “TimeMaxExp, “isTF” and “coded.expr.level”, and allow to pass to the function the criteria to select genes that will be included into the output. As an example, assume that a researcher interested in MYB transcription factors—genes that regulate plant responses^37^, but in particular wants to investigate expression profiles of this kind of genes that have its maximum expression at either, 0 or 20 DAA in the accession “Criollo de Morelos (CM 334)”; see Table 1. Then, the selection of gene sets can be easily performed by the following R commands:

1. temp1<- get.SEP(descr=”MYB”, acc.key=”CM”, TimeMaxExp=0, isTF=TRUE)
2. temp2<- get.SEP(descr=”MYB”, acc.key=”CM”, TimeMaxExp=20, isTF=TRUE)

In both cases the function is call with parameters “descr=”MYB””, which will select only genes that contain within their protein description the chain “MYB”, *and* “acc.key=”CM”” which will select cases that belong to the accession CM, *and* which also fulfill “isTF=TRUE”—which means that the genes are annotated as transcription factors. The differences between the output objects, “temp1” and “temp” is given because in the first we ask for cases where the time where the maximum expression is reached is at 0 DAA (“TimeMaxExp=0”), while in the second case we ask for cases where that point is reached at 20 DAA (“TimeMaxExp=20”).

The output of the function get.SEP(), obtained with statements (1) and (2) above, are SEP data frames. Thus we obtain information of how many different genes fulfill the parameters (in this particular case there are 30 and 12 genes in “temp1” and “temp2”, respectively), as well as the numerical values of the estimated SEPs and extra information about other of the attributes of these genes.

**Figure 3:**
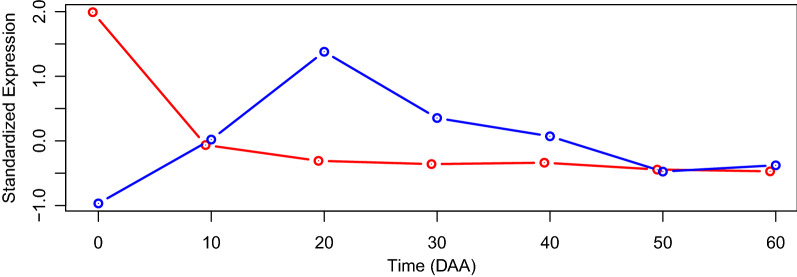
Raw plot resulting of running the function “SEPs.plot(list(temp1, temp2), colors = c(”red”, ”blue”))”. The input (objects “temp1” and “temp1”) are sets of SEPs containing MYB transcription factors expressed in the accession “CM” at times 0 and 20 DAA, respectively.

We can now proceed to the “Summarization” step of the analysis, represented in the second row of rectangles in Fig. 2. Let’s begin by plotting the two sets of SEPs by calling the function SEPs.plot with parameters “SEPs.plot(list(temp1, temp2), colors=c(”red”, ”blue”))”. The direct result of this call is shown in Fig. 3.

**Figure 4:**
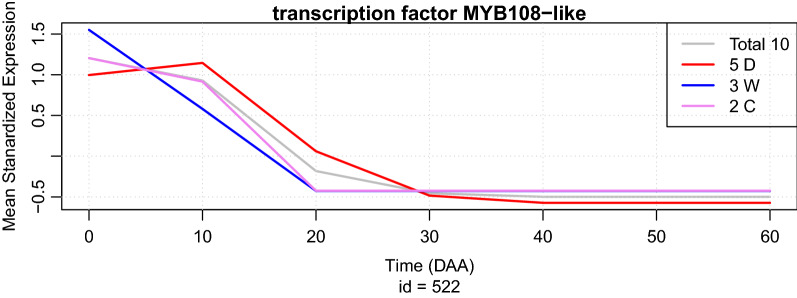
Result of running “gene.summary(temp1$id[1])” (see Text).

Figure 3 shows the raw result of running the function “SEPs.plot()” with main input consisting on the objects “temp1” and “temp1”, which are sets of SEPs containing MYB transcription factors expressed in the accession “CM” at times 0 and 20 DAA, respectively. Additionally, parameter “col” is set to red and blue to determine the colors that will be used in the plot for objects “temp1” and “temp1”, respectively. This plot presents the average of SEPs for the 30 MYB transcription factors with maximum expression at the mature flower (0 DAA)—red line, while the blue line presents the average of SEPs for the 12 MYB transcription factors with maximum expression at 20 DAA, in both cases in accession CM. 95% confidence intervals (CI) are also plot at each time of expression, however, those CI are too narrow to be visible in the scale of the graph. If desired the user can add extra annotations to the plot, as legends of descriptions, by using the corresponding R commands. The summarization step can proceed by examining the summaries of the data frames “temp1” and “temp2”, by calling SEP.summary(temp1) and SEP.summary(temp2), and also by using function “gene.summary” for each one of the genes at each one of the two groups; for example by calling “gene.summary(temp1$id[1])”, produces the plot shown in Fig. 4 of the SEPs in all 3 sets of accessions (D, W and C) as well as the average SEP for the gene with “id=552” that corresponds to a *transcription factor MYB108-like*. Figure 4 presents the plot obtained by this call. The user will also obtain a statistical summary of the expression profiles of this gene in all 12 accessions (not shown).

In Fig. 4, obtained to investigate the expression profile of the first gene of interest (with id 552), the researcher gets information of the average time profiles in all the collection of 12 accessions, and also a statistical summary of those time profiles that for brevity is not shown. The summarization step can also include calling the gene.summary() function in the remaining 41 MYB transcription factors included in “temp1” and “temp2”, and also browsing the NCBI site to obtain detailed information about the proteins coded by these genes using the function “browse.gene()”.

The last step in the data mining workflow in Salsa corresponds to “Analyses” in Fig. 2. It is worth noticing the the corresponding box of this figure repeats the functions “SEPs.plot” and “SEPs.summary” that also appear in the box “Summarization” of that figure. This is so because the mentioned functions give also results about the sets of interest that can be interpreted by the user. Now, by calling the function “analyze.2.SEPs” with parameters “analyze.2.SEPs(temp1, temp2)” the researcher could perform the *t*-test of Euclidean distances between and within the two SEPs, and confirm that, as shown in Fig. 3, the average time profiles are significantly different (*p*-value $< 2.2 \times 10^{-16}$; details of the test not shown). Finally, if desired, the user can perform a full set of GO enrichment analyses by using function “analyze.all.GO” on the genes found in the objects “temp1” and “temp2”.

The simplified workflow schema for Salsa, shown in Fig. 2 is linear, and thus it does not reflects the iterative nature of data mining. In reality, data mining processes are iterative—iteration, by alternating functions, altering sets of interest or focussing into particular aspects are in many cases necessary for the generation of relevant hypotheses^38,39^. Accordingly, the simple example presented above only gives a glance to the Salsa possibilities to find interesting aspects in the time profiles contained in the package. Next section presents a more detailed example.

### Comparing gene expression profiles between accessions with contrasting fruit size

We begin our analysis by isolating, as separate SEP data.frames, the genes expressed in accessions, “AS” (Ancho San Luis), of the domesticated accession set, which produces very large and moderately pungent fruits and “SR” (Sonora Red), a wild accession with very small and highly pungent fruits (see Table 1). R code and details of the analyses in this section are in Additional file 1.

**Figure 5:**
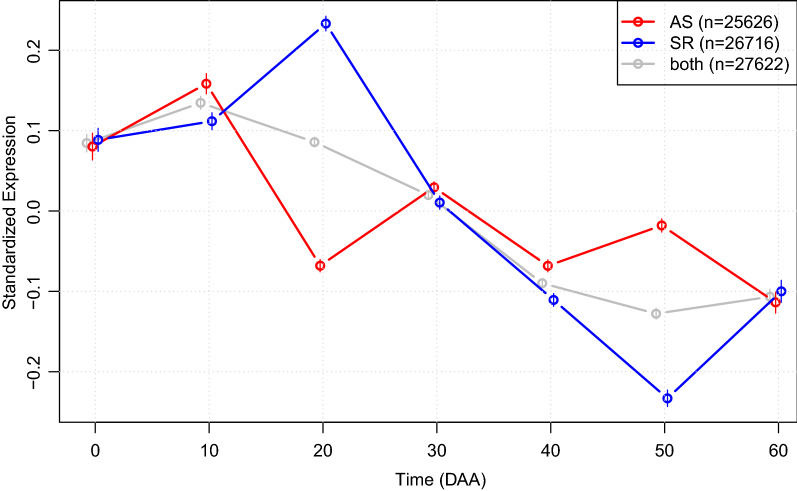
Plot of average Standardized Expression Profiles (SEPs) in groups formed by accessions “AS” (in red), “SR” (in blue) and the SEPs including all genes from both accessions (in grey). Thin vertical lines over the circles marking each mean are the 99.99% ($\alpha = 1 \times 10^{-4}$) confidence intervals (CI’s) for the means. Plot obtained with function “SEPs.plot()”.

The majority, $> 89\%$, of the genes were consistently expressed in both accessions, while small percentages, $< 3\%$ and $< 7\%$ of the total number of genes, were exclusively expressed in “AS” and “SR”, respectively. Figure 5 presents the plot of the average SEPs for each accession, as well as for the set formed by both of them.

Figure 5 shows that the average SEPs in “AS” and “SR” significantly differ at some points of the fruit development. For this plot we employed a very stringent threshold for the estimation of confidence intervals (CI) for the means; an Error Type I of $\alpha = 1 \times 10^{-4}$, which implies a 99.99% of confidence. CIs for the mean of each group at each time are shown as thin vertical lines over the circles that stand for the means per time and accession group. Looking at the CIs, we see that the mean SEPs of “AS” and “SR” are highly different at time points 10, 20, 40 and 50 DAA. It is important to consider that the plot of average SEPs, as the one presented in Fig. 5 for “AS” (red line) and “SR” (blue line), does not indicate uniformity of expression profiles for individual genes; in fact, the mean of the SEPs hides the large diversity of individual expression profiles among genes (see Fig. 3 in Additional file 1).

#### Finding sets of genes with divergent expression between the two accessions

The divergence of average SEP expression between “AS” and “SR”, observed in Fig. 5, entails high differences between the transcriptomes of the two accessions; in particular, the peak of mean expression is found at 10 DAA for “AS”, while for “SR” it happens ten days later, at 20 DAA. Peak of mean expression signals maximum transcriptional activity, and is a hallmark in time for each individual gene.

**Figure 6:**
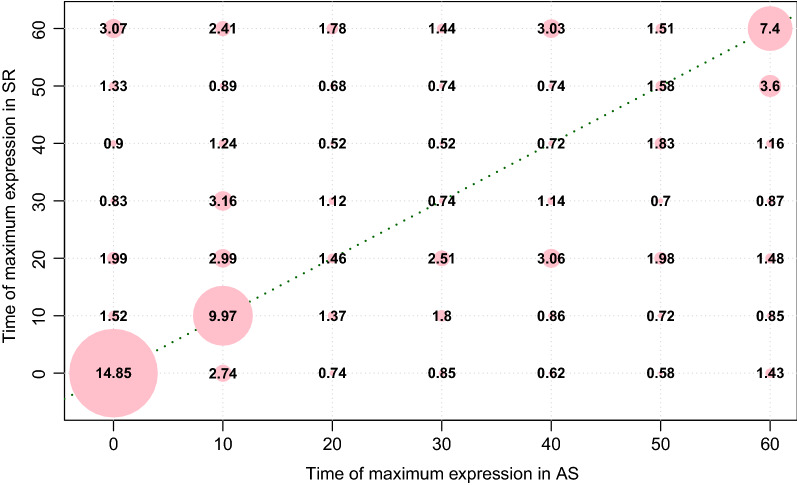
Matrix of percentages of genes peaking at each one of the 49 possible combinations of seven times in accession “AS” (*X*-axis) and seven times in accession “SR” (*Y*-axis). The percentage of genes simultaneously presenting the peaks is shown at each intersection, while the size of the circles at each intersection corresponds with the proportion of genes. The green dashed line in the diagonal signals the cases where the expression peaks coincide in both accessions.

To dissect transcriptome differences between “AS” and “SR”, we selected the sets of genes with simultaneous peak expression at each one of the seven sampled time points. This produces a total of $7 \times 7 = 49$ gene sets (see Box 3 in Additional file 1). Figure 6 shows the matrix of percentages of genes with peak expression at each one of the 49 times combinations.

The total of genes expressed in both accessions was 24720. Of these, 3672, representing a proportion of $3672/24720 \approx 0.1485$ or 14.85%, have their peak expression at 0 DAA in both accessions That figure is presented in the bottom left hand-side of the matrix in Fig. 6. The green dashed line in Fig. 6 signals the cases where the peak expression coincides in time in both accessions, and we can see that, except for 0, 10 and 60 DAA, the corresponding percentages are small (less than 2%), which partially explains the differences between the average SEPs observed in Fig. 5.

Gene sets that are out the dashed green diagonal of Fig. 6 are “interesting”, in the sense that they present a pattern where peak expression are out of phase. One of the two sets of genes presenting the highest possible phase difference is the one formed by the 758 genes ($\approx 3.07\%$ of the total; top left hand-side corner in Fig. 6) which peak at 0 DAA in “AS” (*X*-axis) while having such maximum at 60 DAA in “SR” (*Y*-axis).

#### Analysis of the “ASm0SRm60” gene set

**Figure 7:**
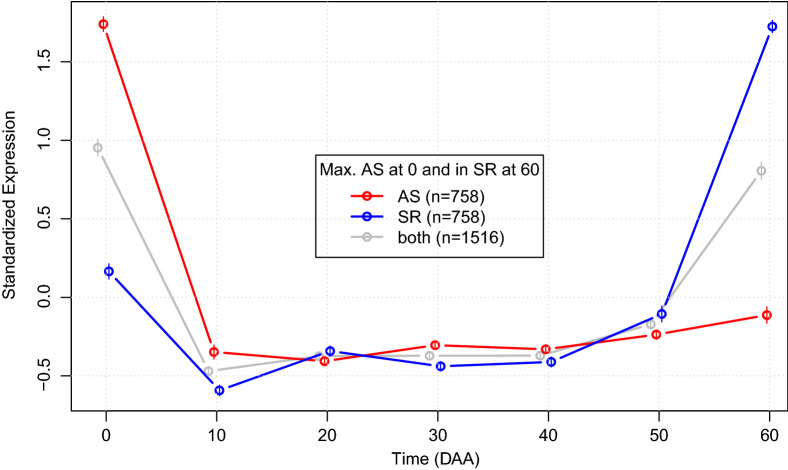
Average expression profiles for genes simultaneously having maximum expression at 0 DAA in accession “AS” but maximum expression at 60 DAA in accession “SR” (set “ASm0SRm60”). Obtained with function “SEPs.plot()”; see Box 4 in Additional file 1.

To further illustrate “*Salsa*” capabilities, we performed an in-depth analysis of the set of 758 genes ($\approx 3.07\%$ of the total), which presents its maximum mean expression at the mature flower (0 DAA) in accession “AS”, while having such peak at the mature fruit (60 DAA) in “SR” (see higher left hand side corner in Fig. 6). We denote that gene set as “ASm0SRm60”. Figure 7 presents the averages of SEPs in the ASm0SRm60 set.

In Fig. 7 we can notice the high difference in phase and contrasting average SEPs in the set ASm0SRm60. Average SEPs in “AS” (red line) presents a high peak at 0 DAA, suddenly decreasing from 0 to 10 DAA and then presenting a relatively steady state from 10 up to 60 DAA, forming an ‘L’ shaped expression profile. On the other hand, average SEPs for “SR” (blue line) presents an almost mirrored L shape with a local maximum at 0 DAA and then decreasing from 0 to 10 DAA—where the global minimum of mean expression is reached. From 20 DAA up to 50 DAA mean expression in “SR” stays relatively steady, suffering then a sudden increase to reach the peak of mean expression at 60 DAA. 53 of the 758 genes in ASm0SRm60 ($\approx 6\%$) are transcription factors (TF), and Fig. 8 in Additional file 1 shows that these genes display a highly significant difference between “AS” and “SR” only at 0 and 60 DAA, showing a low and not significant steady state between 10 and 50 DAA.

The expression pattern of ASm0SRm60 genes is intriguing because it reverses peak expression from the first stage of fruit development—the mature flower at 0 DAA in domesticated accession “AS”, to the last stage—fully mature fruit at 60 DAA in wild accession “SR”. To understand the biological relevance of this set of genes we performed GO enrichment analyses by running function “analyze.all.GO” with a FDR threshold of 10% with categories Biological Process (BP), Cell Component (CC) and Molecular Function (MF). Results are summarized in Table 5.

**Table 5:** Selected Gene Ontology enrichment analyses results.

Category	Id	Description	Odds	O	E
Biological process	GO:0006810	Transport	1.72	106	70
Biological process	GO:0051649	Establishment of localization in cell	2.02	39	21
Biological process	GO:0016236	Macroautophagy	22.48	4	0
Cell component	GO:0009579	Thylakoid	0.00	0	9
Cell component	GO:0009521	Photosystem	0.00	0	8
Cell component	GO:0005778	Peroxisomal membrane	15.72	4	0
Molecular function	GO:0022892	Substrate-specific transporter activity	1.90	42	23

“O” and “E” are Observed and Expected number of genes in the “ASm0SRm60” set, respectively.

In the first row of Table 5 we can see that a total of 106 genes from the set of 758 in “ASm0SRm60”, i.e., $\approx 14\%$, are annotated in the BP ‘Transport’ (GO:0006810), while the expected number of such genes under the independence hypothesis is only 70. This implies that small molecule transport is higher in the mature flower (0 DAA) in the domesticated accession “AS”, while it is higher in the mature fruit (60 DAA) in the wild accession “SR” (see Fig. 7).

We are not going to extend here the discussion of the biological relevance of the results in Table 5; nonetheless, it must be clear that Salsa capabilities grant detailed and deep mining of the chili pepper transcriptome during fruit development in the 12 accessions sampled (see Additional file 1 for more details).

### Extending Salsa algorithms to other datasets

The methodology to estimate and analyze SEPs can be extended to almost any time course experiment, including not only RNA-Seq data, but also other expression profiling methods as microarrays or metabolomic time profiling experiments, as the ones described in^40^. However, SEP estimation is dependent on the specific data type as well as on the number and separation of the times sampled. In summary, to estimate SEPs we need replicated measures of the target data at times that include the whole relevant time period. It is also advisable that sampling times will be equally separated, forming intervals with the same time length. Constructing ternary models, that implies hypothesis testing of neighboring intervals to decide if the variable has not significantly changed (remained at an steady state, “S”), or it increased (“I”) or it decreased (“D”), at each interval is the core of SEPs estimation, and can be performed for any time course experiment, but the particular method for hypothesis testing depends on the nature of the data. Having such ternary model for the variable of interest, the standardization necessary is straightforward, and, as seen here, SEP plotting, grouping and analysis present advantages for the interpretation of the results. A disadvantage of this approach is that SEP estimation use error variance (differences between replicates), and thus further analyses must rely on indirect evidence, as the Euclidean distances between and within SEPs sets. However, this approach is statistically robust giving good practical results, even when it is computationally heavy when SEPs sets are large.

In conclusion, we presented a methodology to summarize time expression profiles directly applicable to any RNA-Seq time course experiment, and which can be adapted to other types of time course experiments. This methodology was applied to a large set of 179 RNA-Seq libraries that estimate gene expression during fruit development in 12 chili pepper accessions. All relevant data and functions to mine these transcriptomes are collected in the Salsa R package, which possibilities for data mining have been demonstrated here. We anticipate that the R package will be useful to the research community studying gene expression changes during fruit development.

### Additional information

The full set of 179 RNA-Seq libraries from which the R package “*Salsa*” originated have been deposited in NCBI’s Gene Expression Omnibus (GEO)^25^, and are accessible through GEO Series accession number GSE165448.

(Link: https://www.ncbi.nlm.nih.gov/geo/query/acc.cgi?acc=GSE165448).

**Salsa R package** The R package “*Salsa*” has been deposited at zenodo and it is publicly available in the link https://zenodo.org/record/4767445#.YKJmFGauJn5 (Salsa at zenodo). On that link you can download the package, its manual as well as instructions to install it.

## Supplementary Information

Supplementary Information.
