# Supplementary material for: A method to analyze time expression profiles demonstrated in a database of chili pepper fruit development
Source: Sci Rep. 2021 Jun 23;11:13181. doi: 10.1038/s41598-021-92672-4 (PMC8222228; doi:10.1038/s41598-021-92672-4)
Supplement: Supplementary file 1 — Supplementary Information. [file 41598_2021_92672_MOESM1_ESM.pdf]

ADDITIONAL FILE 1.
R code and supplementary results for
"A method to analyze time expression profiles demonstrated in a database of chili pepper fruit development"

Christian Escoto-Sandoval, Alan Flores-Diaz, M. Humberto Reyes-Valdés, Neftali Ochoa-Alejo and Octavio Martinez.

Abstract. Here we present the R code and brief explanations of the corresponding results for the examples of data mining presented in the main text of the paper. We focus in the use of "*Salsa*" data and functions and do not interpret the biological relevance of them. Some of the figures presented in the main text are repeated here for continuity, thus this document can be independently used as a vignette for the package.

NOTE: This document was written using "*Salsa*" Version 0.5. The package is available at Zenodo in the link "Salsa: An R package of data mining facilities for *Capsicum* gene expression profiles" Martínez and Escoto-Sandoval (2021).

For comments and complains please mail to: octavio.martinez@cinvestav.mx.

Contents

1. Introduction 1
1.1. The RNA-Seq experiment 1
1.2. The need for a data-mining tool 2
2. Introduction to "*Salsa*" 3
2.1. Data and relational structure 3
2.2. The "`get.SEP`" function. 4
2.3. How many curated data do we have? 8
3. An example of Data Mining: Comparing two Accessions 11
3.1. Isolating sets of interesting genes 19
3.2. Gene Ontology analyses of the "`ASmOSRm60`" set of genes 20
3.3. Transcription Factors in the `ASmOSRm60` gene set 24
3.4. A general summary for a given gene 29
References 30

1. Introduction

1.1. **The RNA-Seq experiment.** The molecular data discussed in this publication, consisting of 179 RNA-Seq libraries, have been deposited in NCBI's Gene Expression Omnibus (Edgar et al., 2002) and are accessible through GEO Series accession number GSE165448:

(https://www.ncbi.nlm.nih.gov/geo/query/acc.cgi?acc=GSE165448).

The full background for the origin and curation of the data in the "*Salsa*" package are detailed in (Martínez et al., 2021).

The experiment from which the data were obtained was a full factorial with two factors: Accessions (or genotypes) at 12 levels and Time in "Days After Anthesis" (DAA). Table 1 presents the 12 accessions used.

Date: June 10, 2021.

Table 1. Accessions (`data.frame` "`acc`" in the Salsa package).

`acc.key`	`acc.type`	`acc.name`
AS	D	Ancho San Luis
CM	D	Criollo de Morelos 334 (CM334)
CO	W	Piquín Coahuila
CQ	C	F_1: CM ♀ × QU ♂
CW	D	California Wonder
JE	D	Jalapeño Espinalteco
QC	C	F_1: QU ♀ × CM ♂
QU	W	Piquín Queretaro
SR	W	Piquín Sonora Red
ST	D	Serrano Tampiqueño 74
SY	W	Piquín Sonora Yellow
ZU	D	Zunla-1

Table 1 is a minor modification to the `data.frame` "`acc`" in the "*Salsa*" package, and it includes columns '`acc.key`' the 'key' for each one of the accessions, '`acc.type`' that classifies accessions into three types, 'D' for 'Domesticated', 'W' for 'Wild' (or 'non-domesticated') and 'C' which denotes the F_1 cross between the D accession 'CM' and the W one 'QU'. 'CQ' is the F_1 resulting from the use of 'CM' as female (♀) and 'QU' as male (♂), while 'QC' is the F_1 of the cross in the other direction, i.e., employing 'QU' as female (♀) and 'CM' as male (♂). Finally column '`acc.name`' gives the common name of the accessions.

The time factor was evaluated every ten days during fruit development, beginning with the mature flower (at 0 DAA) up to the end of the maturation of the fruits at 60 DAA, a point where the fruits were fully mature and for the majority of the accessions they were already entering senescence. For convenience, the vector of the seven times sampled during fruit development is kept into the numeric vector `DAA = c(0, 10, 20, 30, 40, 50, 60)` within the "*Salsa*" package.

The traditional way to evaluate the results of RNA-Seq experiments (Wang et al., 2009) is to perform contrasts between treatment to obtain sets of 'differentially expressed genes'. However, given that the data are from a time course experiment (Spies and Ciaudo, 2015), we decided to summarize the available information as "Standardized Expression Profiles" (SEPs). Briefly, SEPs are seven dimensional numeric vectors in which each one of the numbers summarizes the standardized mean expression at the corresponding time point –obtained from two RNA-Seq libraries (vector `DAA`), and given the standardization the sum of each SEP is equal to 0 and its standard deviation equal to 1. The statistical method employed implies that misclassification of SEPs is approximately of 1% (see details in Martínez et al. (2021)).

1.2. **The need for a data-mining tool.** In traditional pre-genomic era experiments, the number of response variables was generally small. In contrast, whole genome RNA-Seq experiments result in tens of thousands of results per treatment, which correspond to the expression levels of each one of the genes expressed during the course of the experiment in each one of the 'libraries'. In practical terms this data avalanche means that results are a 'mine' of putative discoveries, which could be 'excavated' looking for new and relevant knowledge. In our experiment the number of original data points was approximately of 4.4 millions, plus the genomic *Capsicum* information, which consist in more than a hundred thousands of curated data points (mainly gene identifications and annotations). Thus, aside traditional statistical methods to analyze results, we need to use 'data mining' techniques to detect non obvious facts. Loosely defined, 'data mining' is the process of discovering patterns in large data sets involving methods at the intersection of machine learning, statistics, and database systems (see relevant document here) (Chakrabarti et al., 2006).

We designed the "*Salsa*" R package to provide an auto-contained set of data and functions to mine our transcriptomic results.

2. Introduction to "*Salsa*"

2.1. **Data and relational structure.** Table 2 presents the content of "*Salsa*", which consist in the numeric vector of times, `DAA` (row d.1), and 9 `data.frame` structures, rows d.2 to d.10. There are also 14 functions, presented in rows f.1 to f.14 in that table. To familiarize you with the package, you could run the 'Examples' that appear at the end when you type "`? Salsa`" in your R window, after installing the package and including it in the environment with the R statement "`library(Salsa)`".

Table 2. Data and Functions in "*Salsa*".

Data		
Row	Name	Short Description
d.1	`DAA`	Days After Anthesis (numeric vector).
d.2	`acc`	Accessions, `data.frame`.
d.3	`SEP`	Standardized Expression Profiles (SEPs), `data.frame`.
d.4	`SEP.id`	Summaries of SEPs for each gene expressed, `data.frame`.
d.5	`gene`	Identifiers for genes, `data.frame`.
d.6	`all.GO`	Gene Ontology (GO) annotations available, `data.frame`.
d.7	`GO.annot`	GO annotations, `data.frame`.
d.8	`FPKM.expr`	Expression data per accession and time in FPKM units, `data.frame`.
d.9	`library.desc`	Description of the 179 RNA-Seq libraries in GEO "GSE165448", `data.frame`.
d.10	`readcounts`	Raw number of reads for each gene at each RNA-Seq library, `data.frame`.

Functions		
Row	Name	Short Description
f.1	`get.SEP`	Obtains a SEP data frame from various criteria.
f.2	`SEPs.plot`	Plot mean expression times in SEPs.
f.3	`SEP.summary`	Summary of a SEP dataframe.
f.4	`analyze.2.SEPs`	Test two SEPs trough Euclidean distances.
f.5	`get.ids`	Selects a set of gene identifiers that fulfill a set of criteria.
f.6	`gene.summary`	Graphic and numeric summary of a gene.
f.7	`browse.gene`	Opens an URL in the page corresponding to a gene identifier.
f.8	`get.GO.terms.by.desc`	Finds GO terms that fulfill the input.
f.9	`browse.GO`	Opens an URL in the page corresponding to a GO identifier.
f.10	`analyze.GO`	GO enrichment analysis (single term).
f.11	`analyze.all.GO`	GO enrichment analysis (all terms).
f.12	`get.mat.from.ids`	2 by 2 Contingency Table.
f.13	`expected`	Expected value of a Contingency Table.

Figure 1 presents the relations between `data.frame`s in "*Salsa*". Each orange rectangle represents a `data.frame`, while the lines connecting them represent links produced by the fact that the paired data frames include common variables. Variable `id` (gene identifier, black lines in the figure) exist in `gene`, `SEP`, `SEP.id`, `GO.annot` and `FPKM.expr` and thus information from these data frames can be obtained by using queries that include more than one of the sets. Variables `acc.id` and `acc.type` give a unique key for each accession and the accession type, respectively (see Table 1). These two variables exist in `acc`, `SEP` and `FPKM.expr` and can be employed to obtain information from arbitrary sets of accessions (red links in Figure 1). Finally, variable `aspect.id` (green line in the figure) links data frames `all.GO` and `GO.annot` and allow Gene Ontology (GO) enrichment analyses, as well as segregation of genes related to each GO term. The use of the relational paradigm in "*Salsa*" implies that the user can perform complex queries (directly or *via* the functions) to obtain a wide spectrum of information in an easy and economical way.

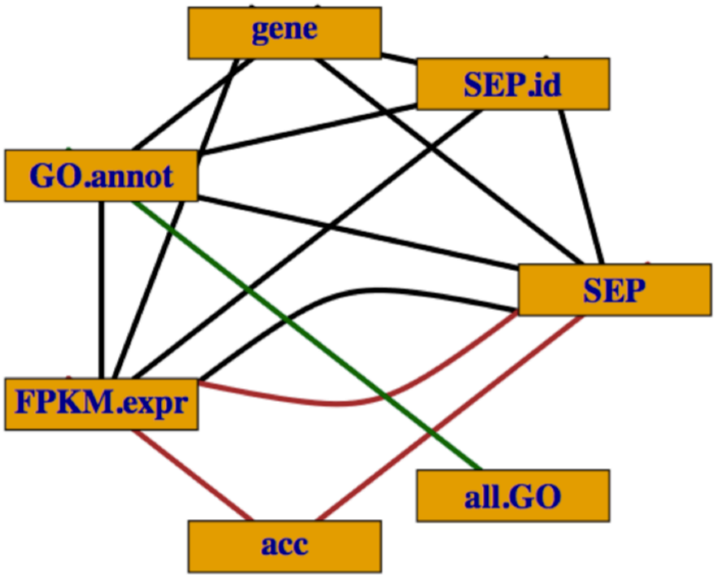

Figure 1. **"*Salsa*" relational structure.** Black = `id`; Red = `acc.id, acc.type`; Green = `aspect.id`. Note data: frames "`library.desc`" and "`readcounts`" are not included in the schema.

Loosely described, the core of the data mining processes that can be performed in "*Salsa*" consist in selecting one or more 'interesting' set of genes in one or more groups of accessions and then perform some analyses on one or more of these gene sets. This simple description hides the number and complexity of putative analyses that could be performed; the number of ways to select sets of genes is immense –we can exaggerate and say 'almost infinite', while the variations in the analyses that could be carried out with each set of 'interesting genes' is also very big.

2.2. **The "`get.SEP`" function.** All operations performed in R will be shown in text boxes. It must be obvious which are R or "*Salsa*" functions (the ones after the prompt '>'), while comments explaining what is going on are given after the '#' sign and R output is shown as it will appear in your command window. If you see an unfamiliar function employed, it will be good idea to employ R help facility, say "> `?  strange.function`" to obtain help.

The core of "*Salsa*" consists in the ability to select particular expression profiles. This task is performed by the "`get.SEP`" function, which select a `SEP data.frame` which will contain rows fulfilling all criterion in the input. Table 3 present the criteria that can be used in "`get.SEP`".

Firstly, it is important to understand that criteria pass to "`get.SEP`" are internally linked by a logical "***and***"; i.e., if criteria 1, 2 and 3 are in the input, then the output will contain cases that fulfill criteria 1 *and* 2 *and* 3. If there are no SEPs which fulfill all criteria in the input, the function will return "`NULL`" (without warning or error). Secondly, within each single criterion (which can consist of a single value or a vector of values), the output is filtered with a logical "***or***"; i.e., if a criterion consist of a vector with three elements, say, e_1, e_2 and e_3, then in the output there will be cases that fulfill e_1 *or* e_2 *or* e_3. This is true for all criteria, except for '`descr`', where elements must fulfill all elements in the vector. A few examples presented below will clarify the meaning of the criteria. In these examples we will only examine the number of rows in the output, which is enough to understand the behavior of the function.

```
--------------------------------------------------------------------------------
### Examples of the use of get.SEP

# 1 - No criteria in the input; thus an error is produced
```

Table 3. Criteria that can be used in the "`get.SEP`" function.

Row	Criterion	Description
1	"`ids`"	(numeric) A subset of "`gene[gene$id]`"; i.e., a numeric vector which elements are valid gene identifiers.
2	"`descr`"	(character) A subset of terms in "`gene[gene$Prot.Desc]`"; i.e., a character chain or vector which appear in some of the protein description within the gene `data.frame`.
3	"`acc.key`"	(character) A subset of "`acc[acc$acc.key]`"; i.e., one or more of the two-letters codes for accession.
4	"`acc.type`"	(character); i.e., any not-empty subset of {"D", "W", "C"}.
5	"`model`"	(character); one or more 6-letters code for a model (see examples below).
6	"`ExistInAll`"	(logic) either "`TRUE`" or "`FALSE`"; if "`TRUE`" only genes that exist in all 12 accessions will be present in the output.
7	"`TimeMaxExp`"	(numeric); any not empty subset of the expression times: $\{0, 10, 20, \cdots, 60\}$. Only SEPs which have their maximum expression at those times will be included in the output.
8	"`isTF`"	(logic) either "`TRUE`" or "`FALSE`"; if "`TRUE`" only genes annotated as coding a Transcription Factor will be part of the output.
8	"`coded.expr.level`"	(numeric); a not empty subset of $\{1, 2, 3, 4, 5\}$ which determines the raw expression level. 1 means very low raw expression and 5 very high raw expression (see "`FPKM.expr`").

```
> get.SEP()
Error in get.SEP() : You need at least one criterion not null to proceed!

# 2 - Using ids (just the first 100 ids that exist in gene)
# See row 1 in Table 3
> temp <- get.SEP(ids=gene$id[1:100])
> nrow(temp)
[1] 1067
> length(unique(temp$id))
[1] 100
# 2.1 Using ids with specific identifiers
> head(gene$id)
[1]  3  6 12 15 17 18
> temp <- get.SEP(ids=c(3, 12, 17))
> nrow(temp)
[1] 25
> unique(temp$id)
[1]  3 12 17

# 3 - Using descr (See row 1 in Table 3)
> temp <- get.SEP(descr="WRKY") # Get genes with "WRKY"
> nrow(temp)
[1] 798
> head(unique(temp$id))
[1]  156  549  604  772 1003 1091
> gene$Prot.Desc[is.element(gene$id, head(unique(temp$id)))]
[1] "probable WRKY transcription factor 69 isoform X1"
[2] "probable WRKY transcription factor 31 isoform X2"
[3] "probable WRKY transcription factor 70"
```

```
[4] "probable WRKY transcription factor 7"
[5] "probable WRKY transcription factor 13"
[6] "LOW QUALITY PROTEIN: WRKY transcription factor 44-like"
# Narrowing the search to:
> temp <- get.SEP(descr=c("WRKY", "69")) # Get genes with "WRKY" AND "69"
> nrow(temp)
[1] 12
> unique(temp$id)
[1] 156
> gene[gene$id==156,]
     id         ProtId                                       Prot.Desc isTF
156 156 XP_016557195.1 probable WRKY transcription factor 69 isoform X1 TRUE

# 4 - Using "acc.key"
# 4.1 SEP with all genes that are expressed in accession "AS"
> temp <- get.SEP(acc.key="AS")
> nrow(temp)
[1] 25626
> unique(temp$acc.key)
[1] "AS"
# 4.2 SEP with all genes that are expressed in accession "AS" OR "QU"
> temp <- get.SEP(acc.key=c("AS", "QU"))
> nrow(temp)
[1] 51847
> unique(temp$acc.key)
[1] "AS" "QU"

# 5 - Using "acc.type"
> temp <- get.SEP(acc.type="C")
> nrow(temp)
[1] 53514
> unique(temp$acc.key) # Only the two accessions that are crosses
[1] "CQ" "QC"

# 6 - Using model
> temp <- get.SEP(model="SSSSSS") # Null model
> nrow(temp)
[1] 10848
> unique(temp$model)
[1] "SSSSSS"
# 6.1 More than one model
> temp <- get.SEP(model=c("SSSSSS", "DSSSSS", "ISSSSS"))
> nrow(temp)
[1] 30512
> table(temp$model) # How many of each one
DSSSSS ISSSSS SSSSSS
 13424   6240  10848

# 7 - More than one criterion at the same time
# 7.1 - Cases of SEP with genes that exist in all 12 accessions
#         and are transcription factors (TFs)
> temp <- get.SEP(ExistInAll=T, isTF=T)
```

```
> nrow(temp)
[1] 18288
> length(unique(temp$id)) # How many genes?
[1] 1524
 # 7.2 - Cases of SEP with genes that exist in all 12 accessions
#         and are transcription factors (TFs) and exist only in
#        accession "AS"
> temp <- get.SEP(ExistInAll=T, isTF=T, acc.key="AS")
> nrow(temp)
[1] 1524
> length(unique(temp$id)) # How many genes?
[1] 1524
# 7.3 - Adding a further requisite, i.e., that the description of the gene
# includes "zinc finger"
> temp <- get.SEP(ExistInAll=T, isTF=T, acc.key="AS", descr="zinc finger")
> nrow(temp)
[1] 210
# 7.4 - Asking also for a very specific model
> temp <- get.SEP(ExistInAll=T, isTF=T, acc.key="AS", descr="zinc finger", model="DISSIS")
> nrow(temp)
[1] 1
> temp
     id acc.key acc.type  model ExistInAll      seT0     seT10      seT20
145 227      AS        D DISSIS       TRUE 0.5417888 -1.609109 -0.4072238
         seT30      seT40    seT50    seT60 TimeMaxExp
145 -0.4072238 -0.4072238 1.144496 1.144496         50
# See the description of that gene (id==227)
> gene[gene$id==227,]
     id         ProtId                           Prot.Desc isTF
227 227 XP_016577631.1 dof zinc finger protein DOF2.1-like TRUE
------------------------------------------------------------------------------------
```

As seen in the previous examples, `get.SEP` is a powerful tool to isolate SEPs, giving a highly flexible way to explore the behavior of the transcriptome. Function '`get.ids`' allows the selection of gene identifiers that fulfill certain criteria. Using both, '`get.ids`' and '`get.SEP`' functions together simplifies the isolation of very specific SEPs, as shown in the examples below.

```
------------------------------------------------------------------------------------
# Obtaining SEP of Example 7.4 (Asking also for a very specific model)
# using get.ids and get.SEP
> ? get.ids
>  temp <- get.SEP(ids=get.ids(ExistInAll=T, isTF=T, descr="zinc finger"), acc.key="AS",
   model="DISSIS")
> temp
     id acc.key acc.type  model ExistInAll      seT0     seT10      seT20
145 227      AS        D DISSIS       TRUE 0.5417888 -1.609109 -0.4072238
         seT30      seT40    seT50    seT60 TimeMaxExp
145 -0.4072238 -0.4072238 1.144496 1.144496         50
> gene[gene$id==227,]
     id         ProtId                           Prot.Desc isTF
227 227 XP_016577631.1 dof zinc finger protein DOF2.1-like TRUE

# Other example:
```

```
# Obtain the ids of all genes that contain the text
# "dof zinc finger protein" in their description:
> temp <- get.ids(descr="dof zinc finger protein")
> length(temp)
[1] 26
> head(gene[is.element(gene$id, temp),])
       id         ProtId                                      Prot.Desc isTF
227   227 XP_016577631.1            dof zinc finger protein DOF2.1-like TRUE
500   500 XP_016574549.1                 dof zinc finger protein DOF3.6 TRUE
2116 2116 XP_016548394.1            dof zinc finger protein DOF2.1-like TRUE
5410 5410 XP_016563618.1                 dof zinc finger protein DOF5.6 TRUE
5506 5506 XP_016542612.1            dof zinc finger protein DOF2.4-like TRUE
7268 7268 XP_016538284.1 dof zinc finger protein DOF2.5-like isoform X2 TRUE
-----------------------------------------------------------------------------------------
```

2.3. **How many curated data do we have?** Let's begin our tour by investigating the extent of the SEP data frame.

```
-----------------------------------------------------------------------------------------
> library(``\emph{Salsa}") # Load the package
# I will assume that you have done that, thus you can repeat the output shown here.

> DAA # The simplest vector (times of expression)
[1]  0 10 20 30 40 50 60

> acc # The accession data.frame (output not shown; see Table 1)

# Now about the SEP data.frame
> ? SEP # To see variable definitions.

> names(SEP) # Contains all SEPs per gene, id, and acc.key
 [1] "id"         "acc.key"    "acc.type"   "model"      "ExistInAll"
 [6] "seT0"       "seT10"      "seT20"      "seT30"      "seT40"
[11] "seT50"      "seT60"      "TimeMaxExp"
> dim(SEP) # Number of rows and columns
[1] 313919     13
-----------------------------------------------------------------------------------------
```

Let's examine in detail a single row of the SEP `data.frame`.

```
-----------------------------------------------------------------------------------------
> SEP[5,] # A row of the SEP data.frame
  id acc.key acc.type  model ExistInAll        seT0    seT10      seT20
5 19      AS        D IDSSSD       TRUE -0.01281868 2.166183 -0.2928162
       seT30      seT40      seT50      seT60 TimeMaxExp
5 -0.2928162 -0.2928162 -0.2928162 -0.9820998         10

# This happens to be the gene with id=19, expressed in the accession acc.key="AS"
# which is of type  acc.type="D" and with model="IDSSSD".

> SEP[5, 6:12] # Columns with standardized expression at each time
         seT0    seT10      seT20      seT30      seT40      seT50      seT60
5 -0.01281868 2.166183 -0.2928162 -0.2928162 -0.2928162 -0.2928162 -0.9820998
```

```
# Note: names "seT<time>" stand for "standardized expression at time <time>".

> mean(as.numeric(SEP[5, 6:12])) # Mean of the SEP (practically 0)
[1] -2.200388e-16
> sd(as.numeric(SEP[5, 6:12])) # Standard deviation (S) of those values
[1] 1
--------------------------------------------------------------------------------
```

Here a brief pause to explain the meaning of the variable '`model`' in the '`SEP`' '`data.frame`'. '`model`' is formed by six concatenated letters, where "D" means a significant decrement in the mean, "S" means that the expression was in a steady (not changing) state and 'I" means a significant increment. The position of the letter within '`model`' gives the corresponding interval, i.e.,

```
--------------------------------------------------------------------------------
> SEP$model[5] # Value of model in the fifth row
[1] "IDSSSD"
> # I = Increment in the first interval; se0 to se10,
> SEP[5, 6:7]
         seT0    seT10
5 -0.01281868 2.166183
> # Second letter in model is D denoting a decrement from time 10 to 20
> SEP[5, 7:8]
     seT10      seT20
5 2.166183 -0.2928162
> # Letters 3 to 5 in model are "S", and the expressions from times 20 to 50 are
> SEP[5, 8:11]
       seT20      seT30      seT40      seT50
5 -0.2928162 -0.2928162 -0.2928162 -0.2928162
> # Finally, the last letter in the model, a "D", corresponds to times 60 to 70:
> SEP[5, 11:12]
       seT50      seT60
5 -0.2928162 -0.9820998
--------------------------------------------------------------------------------
```

Given that we have 3 possible states at each one of the 6 time intervals (D, S or I), the total number of possible models is $3^6 = 729$ and, as we will see later, all of them are present in our data.

Let's now see how many *Capsicum* genes were expressed during fruit development, and are thus included in our data.

```
--------------------------------------------------------------------------------
# The total number of different models present in SEP
> length(unique(SEP$model))
[1] 729
# Number of SEPs in SEP
> nrow(SEP)
[1] 313919
# Number of different genes which has at least one SEP
> length(unique(SEP$id))
[1] 29946
# And we have information for each one of those genes in "gene"
> nrow(gene)
[1] 29946
```

```
# Thus, in average, we have
> 313919/29946 # SEPs per gene
[1] 10.48284
# Some genes were expressed in all 12 accessions, How many?
> length(unique(SEP$id[SEP$ExistInAll==TRUE]))
[1] 22374
# And those genes have
> nrow(SEP[SEP$ExistInAll==TRUE,])
[1] 268488
# SEPs; note that
> 268488/12
[1] 22374

> summary(SEP[,6:13]) # Summary of quantitative SEP variables
      seT0             seT10             seT20             seT30
 Min.   :-2.2678   Min.   :-2.2678   Min.   :-2.2678   Min.   :-2.26778
 1st Qu.:-0.7456   1st Qu.:-0.5191   1st Qu.:-0.3914   1st Qu.:-0.39554
 Median :-0.1010   Median : 0.0000   Median : 0.0000   Median :-0.04551
 Mean   : 0.1414   Mean   : 0.1569   Mean   : 0.1044   Mean   : 0.08502
 3rd Qu.: 1.0690   3rd Qu.: 0.8018   3rd Qu.: 0.5855   3rd Qu.: 0.55609
 Max.   : 2.2678   Max.   : 2.2678   Max.   : 2.2678   Max.   : 2.26779
     seT40              seT50             seT60           TimeMaxExp
 Min.   :-2.26779   Min.   :-2.2678   Min.   :-2.2678   Min.   : 0.00
 1st Qu.:-0.52344   1st Qu.:-0.7795   1st Qu.:-0.9171   1st Qu.:10.00
 Median :-0.26786   Median :-0.3794   Median :-0.3941   Median :20.00
 Mean   :-0.07242   Mean   :-0.2609   Mean   :-0.1543   Mean   :24.78
 3rd Qu.: 0.37796   3rd Qu.: 0.1304   3rd Qu.: 0.4450   3rd Qu.:40.00
 Max.   : 2.26779   Max.   : 2.2678   Max.   : 2.2678   Max.   :60.00

> round(summary(unlist(SEP[,6:12])),2) # Summary of standardized expressions
   Min. 1st Qu.  Median    Mean 3rd Qu.    Max.
  -2.27   -0.59   -0.22    0.00    0.56    2.27

> table(SEP$TimeMaxExp)

    0    10    20    30    40    50    60
76112 64579 35974 34624 28235 21836 52559
> round(100*table(SEP$TimeMaxExp)/313919,2) # In %

    0    10    20    30    40    50    60
24.25 20.57 11.46 11.03  8.99  6.96 16.74

> plot(DAA, 100*table(SEP$TimeMaxExp)/313919,
   ylab="Percentage of SEPs reaching maximum expression")  # Plot not shown
> grid()
> mean(100*table(SEP$TimeMaxExp)/313919) # Mean of % of time of maximum expression
[1] 14.28571
> abline(h=mean(100*table(SEP$TimeMaxExp)/313919), col="red", lty=2)
-----------------------------------------------------------------------------------------
```

From the box above we see that we have information about 29946 different genes, of which 22374 (≈ 75%) were expressed in all 12 accessions. We also see that the standardized expression for all 7 times has

approximate minimum of -2.27 and maximum of 2.27. The variable '`TimeMaxExp`' is the time at which the maximum standardized expression in the SEP was reached. It has a mean of 24.78 and a median of 20, being 0 DAA the time at which more SEPs (24.25%) reached the maximum expression.

3. An example of Data Mining: Comparing two Accessions

Here we document details to obtain the results presented in the section "**Comparing gene expression profiles between accessions with contrasting fruit size**" of the main text.

As an example of "*Salsa*" data mining we will compare two accessions, 'AS', domesticated ('D') and 'SR', a wild ('W') one, classified as *Capsicum annuum* var. *glabriusculum* and which was collected from the Sonora state in Mexico (Hayano-Kanashiro et al., 2016). The phenotypic differences between these two accessions is enormous; "AS" has a very large and bulky fruit mildly pungent, while "SR" has very small round and highly pungent fruits. The first question that comes to mind is if the expression profiles (SEPs) of these two accessions differ. At this point we will be interested only in the set of genes which where expressed in all 12 accessions.

Box 1 presents the commands to obtain the SEPs of genes with expression in the selected accessions and a plot to compare the mean SEP profiles.

Box 1

```
------------------------------------------------------------------------------------------
# > library(Salsa) # If not already done, load package.
# > ? get.SEP # To familiarize with this function.

# Get relevant SEPs containing all rows that include:
# accessions "AS" OR "SR"
> sep.AS.or.SR <- get.SEP(acc.key=c("AS", "SR"))
# accession "AS" only
> sep.AS <- get.SEP(acc.key="AS")
# accession "SR" only
> sep.SR <- get.SEP(acc.key="SR")

# Obtain a summary of those 3 SEPs together:
# ? SEP.summary # To familiarize with this function.
> seps.sum1 <- SEP.summary(sep.AS.or.SR, sep.AS, sep.SR, conf.level=0.9999)
# See what do we have there
> class(seps.sum1)
[1] "list"
> names(seps.sum1)
[1] "general"       "time.means"    "LL.time.means" "UL.time.means"

# See only the first component of seps.sum1

> seps.sum1$general
    n.rows n.ids n.acc n.type n.mod mean.TimeMaxExp LL.TimeMaxExp UL.TimeMaxExp
g.1  52342 27622     2      2   720        26.16465      25.78563      26.54366
g.2  25626 25626     1      1   575        25.06478      24.52453      25.60503
g.3  26716 26716     1      1   716        27.21964      26.68892      27.75036

# Number of genes expressed in both accessions
> length(intersect(sep.AS$id, sep.SR$id))
```

```
[1] 24720

# The other three components contain
# $time.means - Mean expression per time for each SEP.
# $LL.time.means - Lower (confidence) Limit for Mean expression per time for each SEP.
# $UL.time.means - Upper (confidence) Limit for Mean expression per time for each SEP.

# Let's plot a summary of the mean expression of all genes in those 3 SEPs.
# ? SEPs.plot
> SEPs.plot(seps=list(sep.AS.SR, sep.AS, sep.SR),
colors=c("grey", "red", "blue"))
> grid()
> legend("topright", legend=c("AS (n=25626)", "SR (n=26716)",
"both (n=27622)"), pch=1, lw=2, col=c("red", "blue", "grey"))
# SHOWN as Figure 1.
------------------------------------------------------------------------------------
```

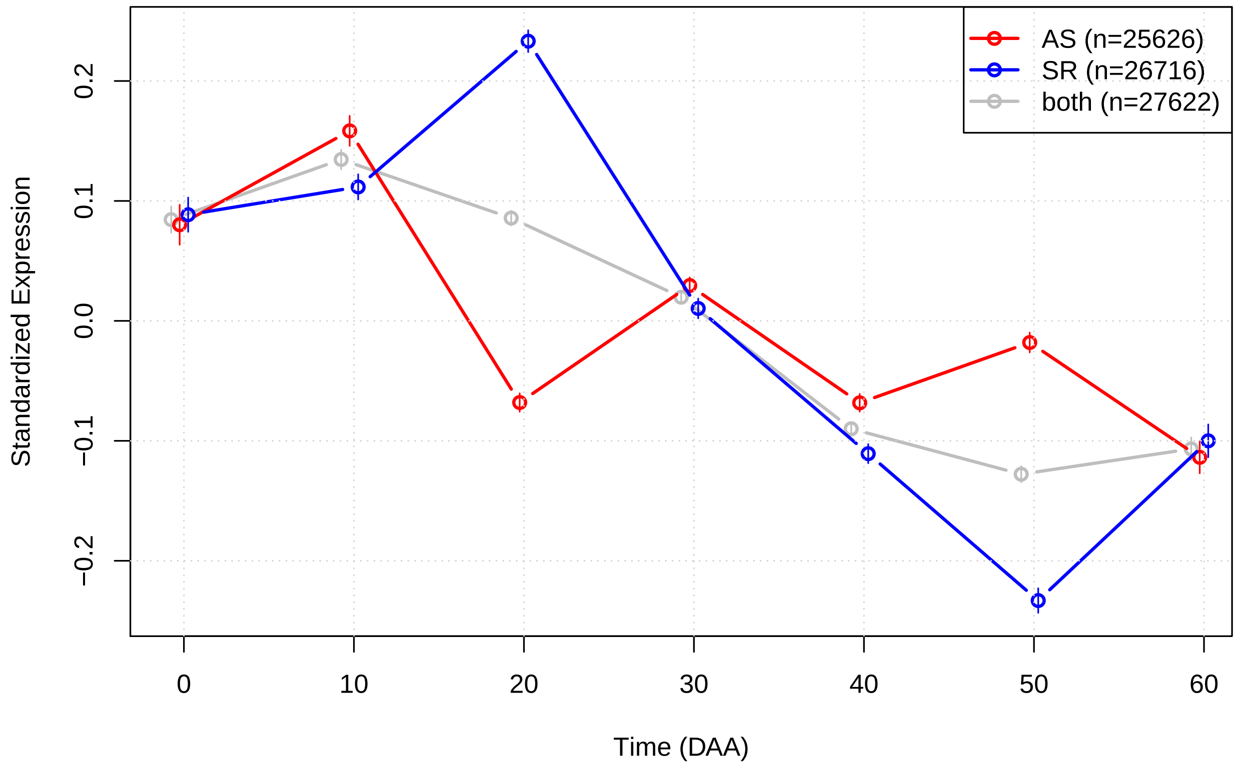

Figure 2. Plot of mean Standardized Expression Profiles (SEPs) in groups formed by accessions 'AS' (in red), 'SR' (in blue) and the SEPs including all genes from both accessions (in grey). Thin vertical lines over the circles marking each mean are the 99.99% ($\alpha = 1 \times 10^{-4}$) confidence intervals (CI's) for the means. Plot obtained with function "`SEPs.plot()`"; see **Box 1**. This figure is presented as "Figure 5" in the main text.

From the first part of the summary of the SEPs in **Box 1** (`seps.sum1$general`), we can notice that the number of genes expressed in "AS" was 25626, while for "SR" it was 26716. The number of genes expressed in either of the two accessions was 27622, while the number of genes expressed in both accessions was 24720, thus a proportion of $24720/27622 \approx 0.8949$ of the genes was expressed in both accessions. On the other hand, in Figure 2 we see that the **means** of the SEPs in the two accessions are remarkable

different at almost all expression times (DAA; X-axis), except at the extremes, 0 and 60 DAA where they are close, and to some extent at 30 DAA, where the two means are also relatively close.

In summary, from Figure 2 we can conclude that the mean SEPs significantly differ between the domesticated "AS" and the wild "SR" accessions. But to reach biologically relevant conclusions we must dissect the SEP enormous differences, grouping genes into 'interesting' subgroups. Before doing that, and to illustrate the amazing heterogeneity of expression patterns we will examine in more detail the SEPs in the "AS" accession (in object "`sep.AS`"). **Box 2** presents some details of the expression of the SEPs and obtains a figure illustrating part of the diversity observed.

Box 2

```
-------------------------------------------------------------------------------------
# A summary of expression per time in sep.AS
> round(apply(sep.AS[,6:12], 2, summary),2)
         seT0 seT10 seT20 seT30 seT40 seT50 seT60
Min.    -2.27 -2.27 -2.27 -2.25 -2.27 -2.27 -2.27
1st Qu. -0.90 -0.50 -0.44 -0.38 -0.45 -0.43 -0.80
Median  -0.12  0.00 -0.19 -0.05 -0.23 -0.19 -0.38
Mean     0.08  0.16 -0.07  0.03 -0.07 -0.02 -0.11
3rd Qu.  1.27  0.77  0.38  0.38  0.38  0.38  0.38
Max.     2.27  2.27  2.27  2.27  2.27  2.27  2.27
> round(apply(sep.AS[,6:12], 2, sd),2)
 seT0 seT10 seT20 seT30 seT40 seT50 seT60
 1.36  1.02  0.63  0.55  0.61  0.68  1.09

# For plotting mean expression
# (could also be obtained from SEP.summary()!)
> temp.mean <- apply(sep.AS[,6:12], 2, mean)

> the.lim <- c(min(sep.AS[,6:12]), max(sep.AS[,6:12]))
[1] -2.267787  2.267787
plot(DAA, temp.mean, type="b", lwd=4, ylim=the.lim,
ylab="Standardized Expression")
grid()
for(i in 1:100){
points(DAA, sep.AS[i,6:12], type="l", col=rainbow(100)[i],
lwd=0.5)
}
# Shown as Figure 2 in text
-------------------------------------------------------------------------------------
```

In **Box 2** we see that for AS, at each one of the seven times, the minimum and maximum of standardized expression has a very large variation that ranges between -2.27 and 2.27, indicating a extensive SEP diversity. Such diversity is shown in Figure 3, by plotting just the first 100 SEPs in "`sep.AS`".

To dissect the differences in expression profiles between "AS" and "SR" we can follow different approaches, for example, we could measure the Euclidean distances between SEPs of each one of the genes expressed in both accessions. This results in distances between 0 and ≈ 4.9 (data not shown), but presents the inconvenience of needing a set of arbitrary thresholds to segregate genes into distance categories. Instead we could use as classification criteria the points at which the gene reaches it maximum expression. This naturally segregate genes in seven categories, one for each one of the times sampled, $0, 10, 20, \cdots, 60$ DAA. We can do that in turn for each one of the two accessions, "AS" and SR, and then we can count

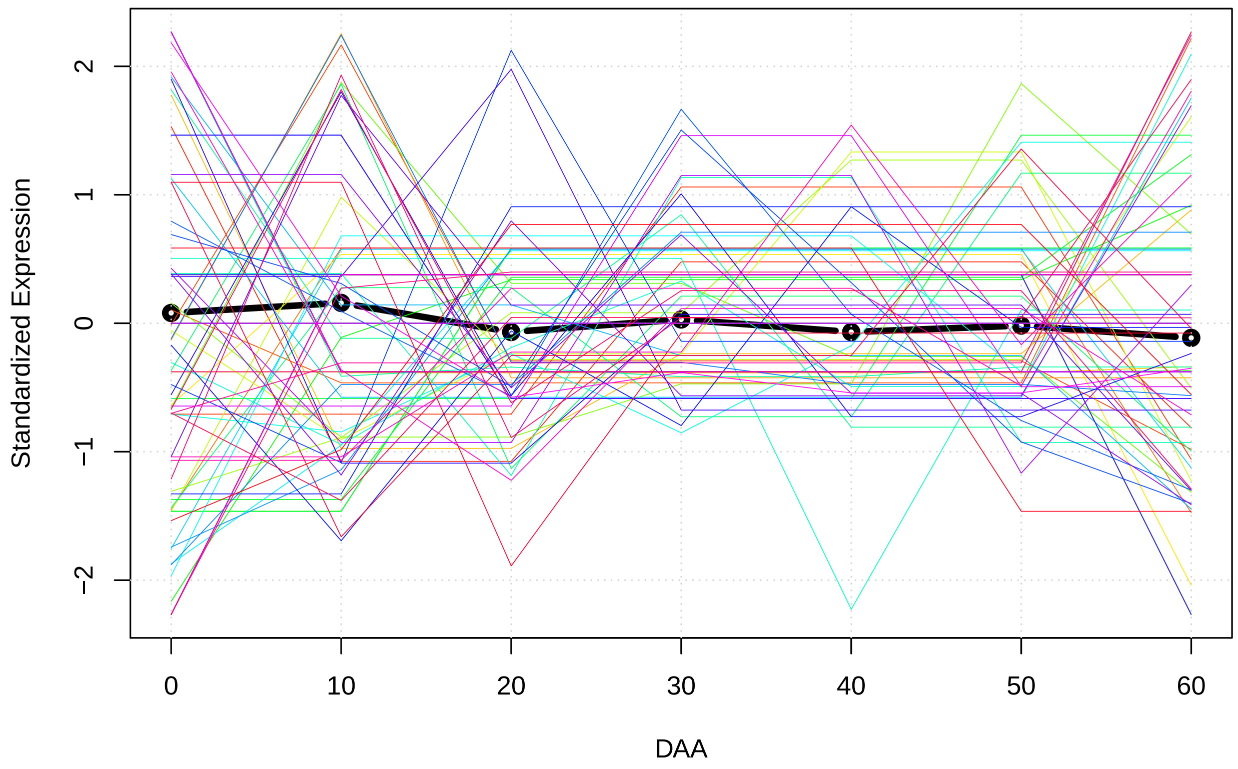

FIGURE 3. Plot of Standardized Expression Profiles (SEPs) in "`sep.AS`". Thick black line presents the mean for the 25626 genes in this SEP (same than red line in Figure 2), while the 100 thin colored lines show the first 100 individual SEPs. See **Box 2**.

how many genes have their maxima at each one of the $7 \times 7 = 49$ possible categories obtained crossing the individual categories from each accession. **Box 3** presents the R computations needed.

Box 3

```
--------------------------------------------------------------------------------
# Obtain seps with genes that have the
# maximum time of expression at each one
# of the seven times sampled (DAA).
> sep.AS.max <- vector("list", 7)
> sep.SR.max <- vector("list", 7)
> names(sep.AS.max) <- paste("sep.AS.m", DAA, sep='.')
> names(sep.SR.max) <- paste("sep.SR.m", DAA, sep='.')

for(i in 1:7){
sep.AS.max[[i]] <- get.SEP(TimeMaxExp=DAA[i], previous.sep=sep.AS)
sep.SR.max[[i]] <- get.SEP(TimeMaxExp=DAA[i], previous.sep=sep.SR)
}

# Obtain the plots of each one of those SEPs
# For AS (presented as Figure 4)
> SEPs.plot(sep.AS.max, colors=rainbow(7), conf.level=0.99999, CIs.sep=0.1)
> text(DAA, y=rep(0.5,6), labels=unlist(lapply(sep.AS.max, nrow)), font=2)
> grid()
```

```
# For SR (presented as Figure 5)
> SEPs.plot(sep.SR.max, colors=rainbow(7), conf.level=0.99999, CIs.sep=0.1)
> text(DAA, y=rep(0.75,6), labels=unlist(lapply(sep.SR.max, nrow)), font=2)
> grid()

# Obtain a data.frame with the number of genes
# (ids) that exist in each one of the sep.AS.max
# and sep.SR.max data.frames as well as in their
# intersections (common genes).
# Note that there are 7*7=49 possibilities
> n.in.seps <- data.frame(m.AS=rep(NA,49), n.AS=rep(NA,49),
     m.SR=rep(NA,49), n.SR=rep(NA,49), n.AS.SR=rep(NA,49))

k <- 0
for(i in 1:7){
for(j in 1:7){
k <- k+1
n.in.seps$m.AS[k] <- DAA[i]
n.in.seps$m.SR[k] <- DAA[j]
n.in.seps$n.AS[k] <- length(sep.AS.max[[i]]$id)
n.in.seps$n.SR[k] <- length(sep.SR.max[[j]]$id)
n.in.seps$n.AS.SR[k] <- length(intersect(sep.AS.max[[i]]$id, sep.SR.max[[j]]$id))
}
}

# Second part:
# Obtains a plot showing the percentages of different
# genes which have their maxima concurrently in
# both of the expression times.

# Total of the number of genes expressed in AS, SR:
> sum(n.in.seps$n.AS.SR)
[1] 24720

plot(x=30, y=30, pch=19, col="white", cex=0, xlim=c(-2,60), ylim=c(-5.5,62),
     xlab="Time of maximum expression in AS",
     ylab="Time of maximum expression in SR")
grid()
for(i in 1:49){
points(x=n.in.seps$m.AS[i], y=n.in.seps$m.SR[i], pch=19,
     col="pink", cex=75*n.in.seps$p.AS.SR[i])
text(x=n.in.seps$m.AS[i], y=n.in.seps$m.SR[i],
     labels=round(100*n.in.seps$n.AS.SR[i]/24720, 2),
     cex=0.8, font=2)
}
abline(a=0, b=1, col="green", lty=3)
# (Shown in Figure 6)
-------------------------------------------------------------------------------------
```

In **Box 3** we obtained SEPs that segregated the genes expressed in accession "AS" into seven disjoint sets of genes which have their maximum expression at $0, 10, \cdots, 60$ DAA (list "`sep.AS.max`"), and did the same for genes expressed in accession "SR" (list "`sep.SR.max`"), plotting the mean expression of the

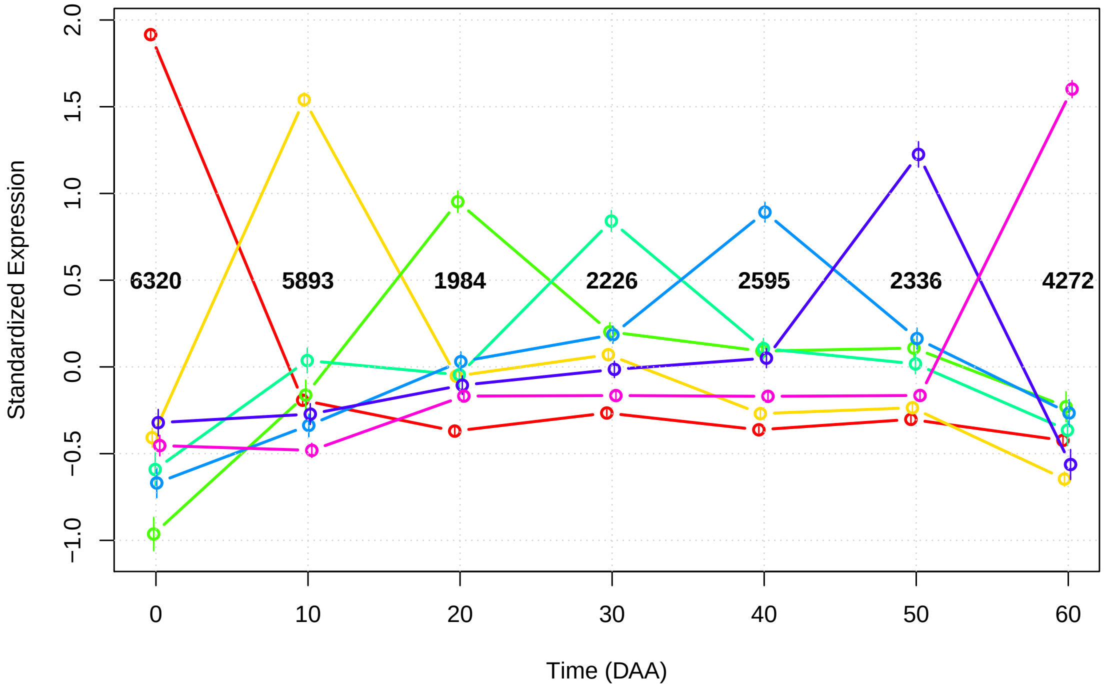

Figure 4. Plot of the 7 SEPs from accession "AS" that segregate genes by their point of maximum expression at $0, 10, \cdots, 60$ DAA shown in different colors. The number of genes in each SEP is shown at $Y = 0.5$

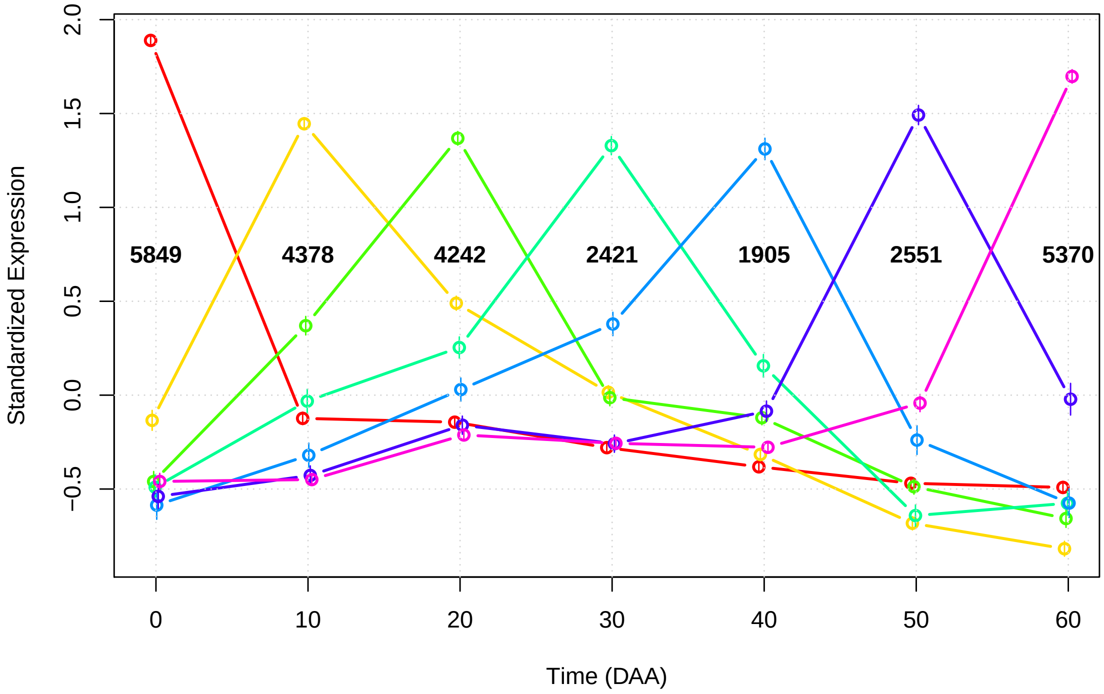

Figure 5. Plot of the 7 SEPs from accession "SR" that segregate genes by their point of maximum expression at $0, 10, \cdots, 60$ DAA shown in different colors. The number of genes in each SEP is shown at $Y = 0.75$

SEPs in figures 4 and 5 for "AS" and SR, respectively. Those figures also show the number of genes that conform each one of the 7 segregated groups.

The results presented in figures 4 and 5 for "AS" and "SR" imply that there are well defined sets of different genes peaking at each one of the 7 sampled times, and these sets differ both, in the number of genes presenting the behavior as well as in the height (Y-axis) at which the peak was reached. In general, the mean profiles of the SEPs presented in figures 4 and 5 is to have a high and well defined maximum at the selected time, but present a relatively steady state in the other time points. For both accessions (AS and SR) the largest (in number of genes) and highest (by height of the peaks) SEPs, are for genes that peak at 0 DAA, with 6320 genes in "AS" (red line in Figure 4) that represent approximately the 25% of all 25626 genes expressed in that accession (6320/25626 = 0.2466), while in the "SR" accession that group is formed by 5849 genes (red line in Figure 5) that represent approximately the 22% of all the 26716 genes expressed in that accession (5849/26716 = 0.2189). These genes present a peak of expression at the mature flower (0 DAA) and much lower expression during the development of the fruit, from 10 up to 60 DAA.

In the second part of **Box 3** we obtained the SEPs of genes that simultaneously present a peak at each one of the $7 \times 7 = 49$ possible combinations of times, and the matrix with the percentages of genes for each one of the intersections is presented as Figure 6.

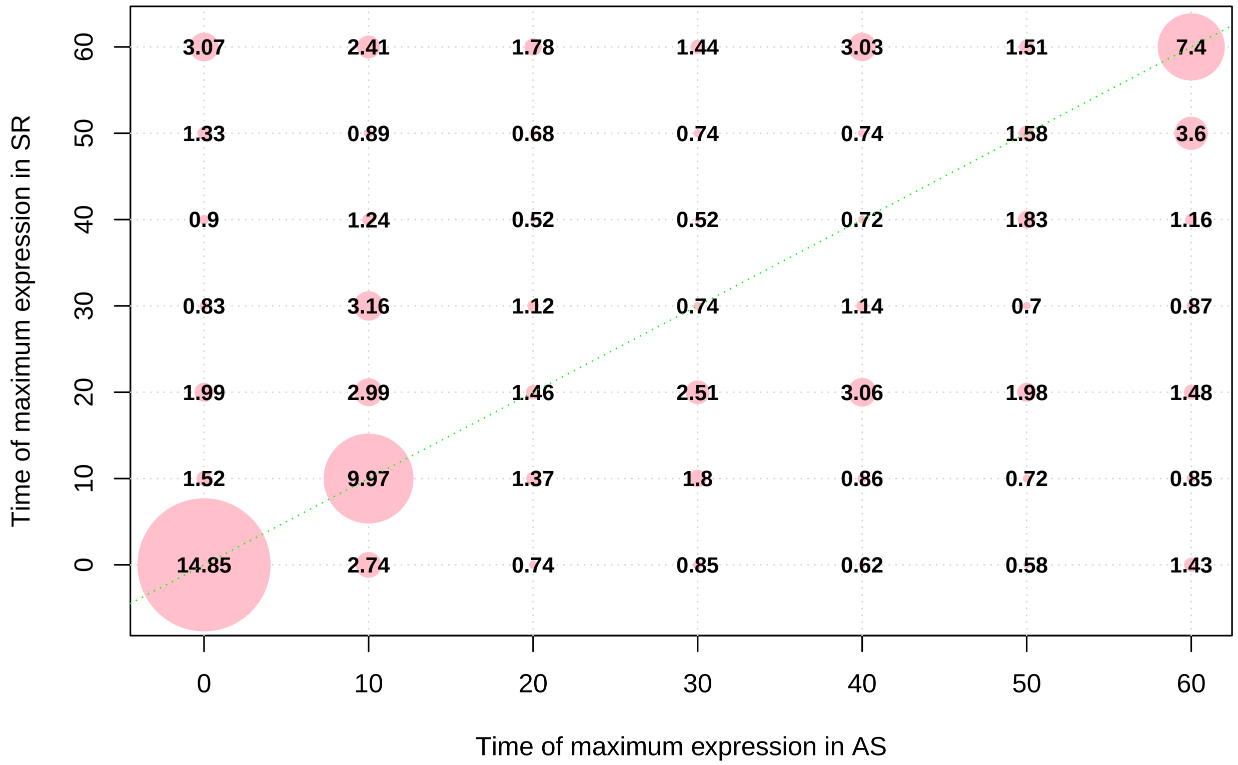

FIGURE 6. Matrix presenting percentages of genes peaking at each one of the 49 possible combinations of seven times in accession "AS" (X-axis) and seven times in accession "SR" (Y-axis). The percentage of genes simultaneously presenting the peaks is shown at each intersection, while the size of the circles at each intersection is proportional to the corresponding proportion of genes. The green dashed line at the diagonal presents the proportion of genes with identical peak in both accessions. This figure is presented as "Figure 6" in the main text.

In Figure 6 the diagonal marked with a dashed green line, and going from the lower left hand side corner to the upper right hand side corner, shows the percentages of genes that coincide in having the maximum

at the same times in both accessions; the raw numbers of genes in that diagonal can be seen in **Box 4**. Genes that present identical peak in both accessions, are consistent in having the maximum expression at both accessions in the same time. In contrast, genes with divergent maximum expression –all those which percentages are out of the diagonal, are "*interesting*" because they diverge between them in the points where the maximum expression was reached, and the degree of divergence is proportional to the distance from the diagonal.

In **Box 4** we show how to isolate SEPs with genes at each one of the 49 combinations shown in Figure 6 for posterior study.

Box 4

```
------------------------------------------------------------------------------------------
# Order n.in.seps by number of common genes
# (genes in the intersection)
> n.in.seps.o <- n.in.seps[order(n.in.seps$n.AS.SR, decreasing=T),]
# Total number of genes that exist in both accessions
> sum(n.in.seps.o$n.AS.SR)
[1] 24720

# Adding a column with the proportion of shared genes
> n.in.seps.o <- data.frame(n.in.seps.o, p.AS.SR=n.in.seps.o$n.AS.SR/24720)

> head(n.in.seps.o)
   m.AS n.AS m.SR n.SR n.AS.SR    p.AS.SR
1     0 6320    0 5849    3672 0.14854369
9    10 5893   10 4378    2464 0.09967638
49   60 4272   60 5370    1829 0.07398867
48   60 4272   50 2551     889 0.03596278
11   10 5893   30 2421     781 0.03159385
7     0 6320   60 5370     758 0.03066343
> tail(n.in.seps.o)
   m.AS n.AS m.SR n.SR n.AS.SR     p.AS.SR
39   50 2336   30 2421     173 0.006998382
20   20 1984   50 2551     168 0.006796117
29   40 2595    0 5849     154 0.006229773
36   50 2336    0 5849     143 0.005784790
19   20 1984   40 1905     129 0.005218447
26   30 2226   40 1905     128 0.005177994

# Summary of number of genes in intersections
> summary(n.in.seps.o$n.AS.SR)
   Min. 1st Qu.  Median    Mean 3rd Qu.    Max.
  128.0   206.0   338.0   504.5   491.0  3672.0
# And in proportions
> summary(n.in.seps.o$p.AS.SR)
    Min.  1st Qu.   Median     Mean  3rd Qu.     Max.
0.005178 0.008333 0.013673 0.020408 0.019862 0.148544

# Number and proportions of genes with identical peak
> n.in.seps.o[n.in.seps.o$m.AS==n.in.seps.o$m.SR,]
   m.AS n.AS m.SR n.SR n.AS.SR     p.AS.SR
1     0 6320    0 5849    3672 0.148543689
```

```
9    10 5893   10 4378    2464 0.099676375
49   60 4272   60 5370    1829 0.073988673
41   50 2336   50 2551     390 0.015776699
17   20 1984   20 4242     360 0.014563107
25   30 2226   30 2421     184 0.007443366
33   40 2595   40 1905     179 0.007241100

# Some of the "Interesting" sets of genes
> head(n.in.seps.o[n.in.seps.o$m.AS!=n.in.seps.o$m.SR,])
   m.AS n.AS m.SR n.SR n.AS.SR    p.AS.SR
48   60 4272   50 2551     889 0.03596278
11   10 5893   30 2421     781 0.03159385
7     0 6320   60 5370     758 0.03066343
31   40 2595   20 4242     757 0.03062298
35   40 2595   60 5370     748 0.03025890
10   10 5893   20 4242     740 0.02993528

# It will be necessary to have all 7*7=49 SEPs with each one of the combinations
# of maximum expression
# define a list for each one of the 7*7=49 SEPS
seps.AS.SR <- vector("list", 49)

k <- 0
for(i in 1:7){
for(j in 1:7){
k <- k+1
temp.AS <- get.SEP(TimeMaxExp=DAA[i], previous.sep=sep.AS)
temp.SR <- get.SEP(TimeMaxExp=DAA[j], previous.sep=sep.SR)
temp.ids <- intersect(temp.AS$id, temp.SR$id)
names(seps.AS.SR)[k] <- paste("ASm", DAA[i], "SRm", DAA[j], sep='')
seps.AS.SR[[k]] <- get.SEP(ids=temp.ids, acc.key=c("AS", "SR"))
}
}
> head(names(seps.AS.SR))
[1] "ASm0SRm0"  "ASm0SRm10" "ASm0SRm20" "ASm0SRm30" "ASm0SRm40" "ASm0SRm50"

# Plot for ASm0SRm60 genes [Figure 6].
SEPs.plot(list(seps.AS.SR$ASm0SRm60,
get.SEP(acc.key="AS", previous.sep=seps.AS.SR$ASm0SRm60),
get.SEP(acc.key="SR", previous.sep=seps.AS.SR$ASm0SRm60)),
colors=c("grey", "red", "blue"))
grid()
legend("center", title="Max. AS at 10 and in SR at 60",
legend=c("AS (n=758)","SR (n=758)","both (n=1516)"),
pch=1, lwd=2, col=c("red", "blue", "grey"))
-------------------------------------------------------------------------------------
```

3.1. **Isolating sets of interesting genes.** As mentioned above, all the $49 - 7 = 42$ sets of genes that have different maximum expression in the two accessions are interesting to be analyzed for a better understanding of the differences in expression between the two accessions. One of the more interesting set of genes is the one that presents maximum expression at 0 DAA in "AS" while presenting the maximum expression at the other extreme of the sampled times, 60 DAA in SR. This set is constituted by 758

genes, representing approximately the 3.07% of all the 24720 genes with expression in both accessions (percentage presented in the upper left hand side corner in Figure 6). Here we denote that set of genes as "`ASm0SRm60`". The mean expression profiles of genes in set "`ASm0SRm60`" are presented in Figure 7.

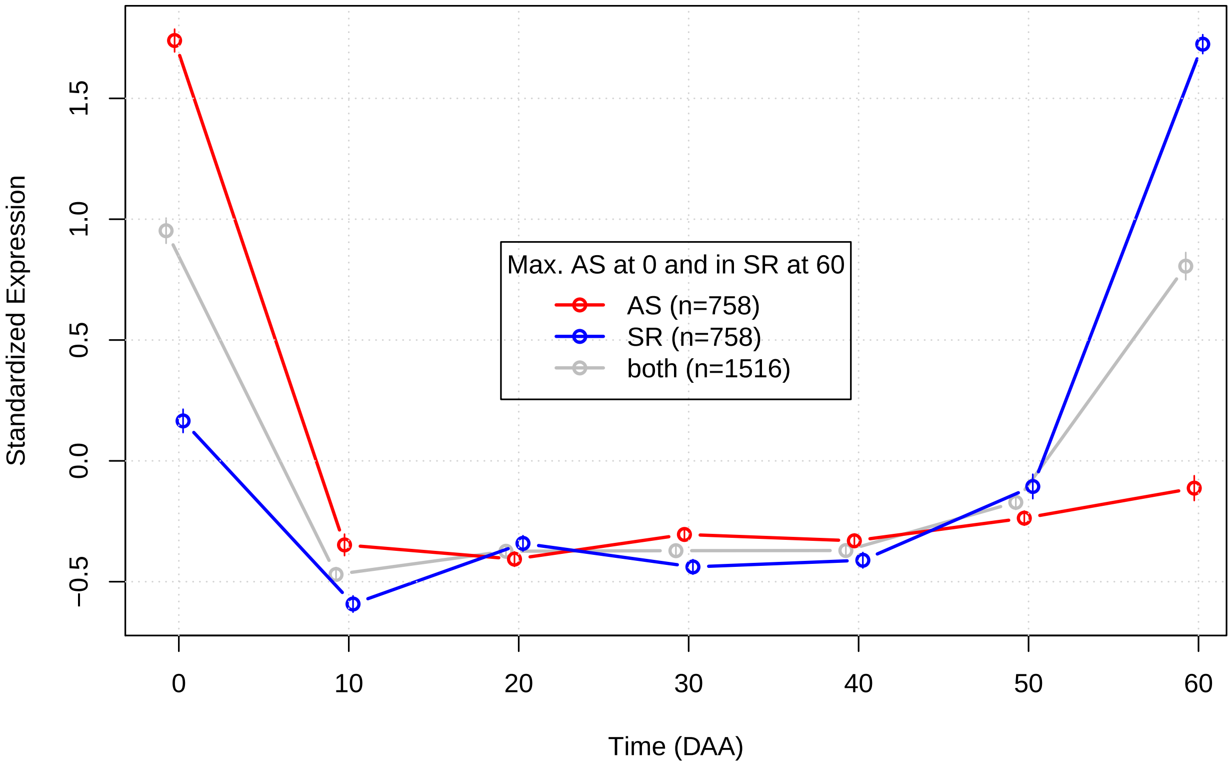

FIGURE 7. Expression profiles for genes simultaneously having the maximum expression at 0 DAA in accession "AS" but maximum expression at 60 DAA in accession "SR" (set "`ASm0SRm60`"). Obtained with function "`SEPs.plot()`"; see **Box 4**. This figure is presented as "Figure 7" in the main text.

In Figure 7 we can see that the mean expression profile of the set "`ASm0SRm60`" in "AS" (red line) has an "L" shape, with relatively small variations in times > 0 DAA, while the expression pattern in "SR" (blue line) is almost exactly opposite to the one for AS, presenting the shape of an specular "L", with relatively small variations for < 60 DAA. The conjoint expression pattern in both accessions (grey line in In Figure 7) presents an "U" shape, with local maxima at 0 and 60 DAA and relatively small mean variation within 10 and 50 DAA.

3.2. **Gene Ontology analyses of the "`ASm0SRm60`" set of genes.** The behavior of the 758 genes having maximum expression at the mature flower (0 DAA) in the domesticated accession AS, while presenting such maximum expression at the fully mature fruit (60 DAA) in the wild accession SR, is intriguing. What are those genes? In which biological process, cell components or molecular functions are they involved? **Box 5** presents the R code aimed to answer those questions.

Box 5

```
--------------------------------------------------------------------------------
# Isolate the ids of the genes in set ASm0SRm60
> ids.ASm0SRm60 <- unique(seps.AS.SR$ASm0SRm60$id)
> length(ids.ASm0SRm60)
[1] 758
```

```
# Perform GO enrichment analyses for genes in set ASmOSRm60.
> ? analyze.all.GO # To familiarize with that function.

# Analysis for Biological Processes (BPs)
GO.BP.ASmOSRm60 <- analyze.all.GO(ids=ids.ASmOSRm60,
     aspect = "BP", only.FDR.le=0.1)

# Analysis for Cell Components (CCs)
GO.CC.ASmOSRm60 <- analyze.all.GO(ids=ids.ASmOSRm60,
     aspect = "CC", only.FDR.le=0.1)

# Analysis for Molecular Function (MFs)
GO.MF.ASmOSRm60 <- analyze.all.GO(ids=ids.ASmOSRm60,
     aspect = "MF", only.FDR.le=0.1)

# Number of GO terms in the results
nrow(GO.BP.ASmOSRm60); nrow(GO.CC.ASmOSRm60); nrow(GO.MF.ASmOSRm60)
[1] 6
[1] 10
[1] 5

# Summary of results for discussion:
> GO.BP.ASmOSRm60[,c(3,4,10)]
                                    desc      odds          Q
412                            transport  1.717503 0.01314055
2053         establishment of localization  1.704101 0.01314055
2037                        localization  1.654068 0.02603585
6                   autophagosome assembly 22.483161 0.05026465
983                      macroautophagy 22.483161 0.05026465
2111 establishment of localization in cell  2.016171 0.06160166
> GO.CC.ASmOSRm60[,c(3,4,10)]
                                         desc     odds          Q
197                                 thylakoid  0.00000 0.02014882
449                            thylakoid part  0.00000 0.02014882
368                    photosynthetic membrane  0.00000 0.02014882
111                       peroxisomal membrane 15.72237 0.02014882
112  integral component of peroxisomal membrane 15.72237 0.02014882
314 intrinsic component of peroxisomal membrane 15.72237 0.02014882
330                         microbody membrane 15.72237 0.02014882
451                             microbody part 15.72237 0.02014882
452                           peroxisomal part 15.72237 0.02014882
191                                photosystem  0.00000 0.02266857
> GO.MF.ASmOSRm60[,c(3,4,10)]
                                                    desc       odds           Q
519                        cysteine-type peptidase activity 0.09852347 0.001886613
1212                                         ADP binding 0.23991135 0.074956239
1034                substrate-specific transporter activity 1.90192953 0.098280260
1246                           transition metal ion binding 1.65126032 0.098280260
1033 substrate-specific transmembrane transporter activity 1.92746090 0.098280260
--------------------------------------------------------------------------------
```

In **Box 5** we obtained 6 BPs, 10 CCs and 5 MFs which frequency is significantly different in genes that belong to the `ASmOSRm60` set. We employed a threshold of maximum of 10% in False Discovery Rate (FDR;`only.FDR.le=0.1` in the function call), but this is only an initial brink, because we need to take into account that the set of results obtained are not independent. In the results the `Q` value is the transformation of the `P`-value obtained in the Fisher's exact test which allows to set the FDR threshold. To fully understand and discuss the meaning of GO enrichment analysis it is convenient to see the details of the most significant results at each one of the GO aspects. Those details are presented in **Box 6**.

Box 6

```
--------------------------------------------------------------------------------------
# Details of the most significant results
> ? analyze.GO # To see function details

# See the most significant of the BPs
> GO.BP.ASmOSRm60[1,]
    aspect aspect.id      desc     odds           P AnnTarg NotAnnTarg AnnNotTarg
412     BP       412 transport 1.717503 7.19271e-06     106        284       3100
    NotAnnNotTarg          Q
412         14265 0.01314055
# and repeat the analysis with analyze.GO
> analyze.GO(ids.ASmOSRm60, aspect="BP", aspect.id=412)
Aspect: "BP"; Aspect id = 412
Description: "transport"
Number of ids in target = 758

Observed matrix:
           Target. Not.Target
Annot.         106       3100
Not.Annot.     284      14265

Rounded expected values:
           Target. Not.Target
Annot.       70.42    3135.58
Not.Annot.  319.58   14229.42

Estimated odds ratio from the table = 1.717503

Fisher's Exact Test for Count Data

data:  temp.t
p-value = 7.193e-06
alternative hypothesis: true odds ratio is not equal to 1
95 percent confidence interval:
 1.356083 2.161866
sample estimates:
odds ratio
  1.717439

# See the most significant of the CCs
> GO.CC.ASmOSRm60[1,]
    aspect aspect.id      desc odds            P AnnTarg NotAnnTarg AnnNotTarg
197     CC       197 thylakoid    0 0.0001838063       0        223        357
```

```
    NotAnnNotTarg          Q
197          8261 0.02014882
# and repeat the analysis
> analyze.GO(ids.ASmOSRm60, aspect="CC", aspect.id=197)
Aspect: "CC"; Aspect id = 197
Description: "thylakoid"
Number of ids in target = 758

Observed matrix:
           Target. Not.Target
Annot.           0        357
Not.Annot.     223       8261

Rounded expected values:
           Target. Not.Target
Annot.           9        348
Not.Annot.     214       8270

Estimated odds ratio from the table = 0

Fisher's Exact Test for Count Data

data:  temp.t
p-value = 0.0001838
alternative hypothesis: true odds ratio is not equal to 1
95 percent confidence interval:
 0.000000 0.388066
sample estimates:
odds ratio
         0

# See the most significant of the MFs
> GO.MF.ASmOSRm60[1,]
    aspect aspect.id                            desc       odds            P
519     MF       519 cysteine-type peptidase activity 0.09852347 1.338024e-06
    AnnTarg NotAnnTarg AnnNotTarg NotAnnNotTarg           Q
519       2        479        882         20812 0.001886613
# and repeat the analysis
> analyze.GO(ids.ASmOSRm60, aspect="MF", aspect.id=519)
Aspect: "MF"; Aspect id = 519
Description: "cysteine-type peptidase activity"
Number of ids in target = 758

Observed matrix:
           Target. Not.Target
Annot.           2        882
Not.Annot.     479      20812

Rounded expected values:
           Target. Not.Target
Annot.       19.17     864.83
Not.Annot.  461.83   20829.17
```

```
Estimated odds ratio from the table = 0.09852347

Fisher's Exact Test for Count Data

data:  temp.t
p-value = 1.338e-06
alternative hypothesis: true odds ratio is not equal to 1
95 percent confidence interval:
 0.01186151 0.35894847
sample estimates:
odds ratio
0.09850416
-----------------------------------------------------------------------------------------
```

In **Box 6** we see that for the BP "transport" the observed number of genes annotated in such aspect in the `ASm0SRm60` set is of 106, while the number of expected annotated genes is only 70.42, thus there is an 'excess' of approximately 1.5 times more observed than expected annotated genes, producing an estimate odd ratio of ≈ 1.72, which gives a highly significant value of 7.193×10^{-6} in the Fisher's exact test.

In the case of the most significant CC we have that the most significant term, "thylakoid" (the photosynthetic membrane), is reduced in the `ASm0SRm60` set; while the observed number of genes is 0, the expected by pure chance is 9, and this depletion is highly significant, having a p-value of ≈ 0.0002

The most significant MF is "cysteine-type peptidase activity", with a number of observed genes equal to 2, while the expected value is 19.17 (odds ratio ≈ 0.0985, significantly different of the neutral value of 1); i.e., as in the case of the most significant CC, the most significant MF is depleted of genes for "cysteine-type peptidase activity", and such reduction is significant with a p-value of 1.338×10^{-6}.

3.3. **Transcription Factors in the `ASm0SRm60` gene set.** Given that the aim here is only to show "*Salsa*" data mining possibilities, we are not going to present the biological implications of the results. However, in the next part we briefly explore the number and nature of the Transcription Factors (TFs) present in the `ASm0SRm60` gene set, and show the functions to browse in the internet GO terms at www.informatics.jax.org and particular genes at NCBI gene. R code is presented in **Box 7**.

Box 7

```
-----------------------------------------------------------------------------------------
# TFs in ASm0SRm60
# Get the relevant SEP
> sep.ASm0SRm60.TF <- get.SEP(isTF=TRUE, previous.sep=seps.AS.SR$ASm0SRm60)
# An alternative way to obtain the same SEP is given by
# > temp <- get.SEP(ids=ids.ASm0SRm60, acc.key=c("AS", "SR"), isTF=TRUE)
# > nrow(temp)
# [1] 106
> nrow(sep.ASm0SRm60.TF)
[1] 106
> ids.ASm0SRm60.TF <- unique(sep.ASm0SRm60.TF$id)
> length(ids.ASm0SRm60.TF)
[1] 53

# Plot of the TFs in ASm0SRm60
SEPs.plot(list(sep.ASm0SRm60.TF, get.SEP(acc.key="AS",
previous.sep=sep.ASm0SRm60.TF), get.SEP(acc.key="SR",
```

```
previous.sep=sep.ASm0SRm60.TF)), colors=c("grey", "red", "blue"))
legend("center", title="TFs; Max. AS at 0 and in SR at 60",
legend=c("AS (n=53)","SR (n=53)","both (n=106)"), pch=1,
lwd=2, col=c("red", "blue", "grey"))
# Presented as "Figure 7" in the text.

# Proportion of TFs in set ASm0SRm60
> length(ids.ASm0SRm60)
[1] 758
> 54/758
[1] 0.07124011
# Proportion of TFs in the whole gene set.
> length(gene$id[gene$isTF==TRUE])/length(gene$id)
[1] 0.06074267

# Isolate gene information for ids.ASm0SRm60.TF
> genes.ASm0SRm60.TF <- gene[is.element(gene$id, ids.ASm0SRm60.TF),]

# Let's see some of the gene descriptions
> head(genes.ASm0SRm60.TF$Prot.Desc)
[1] "zinc finger protein ZAT11-like"
[2] "transcription factor bHLH87 isoform X2"
[3] "zinc finger CCCH domain-containing protein 30-like isoform X1"
[4] "transcription factor MYB1R1"
[5] "zinc finger CCCH domain-containing protein 30-like"
[6] "bZIP transcription factor 53"
> tail(genes.ASm0SRm60.TF$Prot.Desc)
[1] "agamous-like MADS-box protein AGL103"
[2] "AT-hook motif nuclear-localized protein 28-like"
[3] "heat stress transcription factor A-3"
[4] "zinc finger protein CONSTANS-LIKE 9"
[5] "B-box zinc finger protein 21-like"
[6] "transcription factor TGA2"

# Let's isolate the genes in "genes.ASm0SRm60.TF"
# which are annotated in the most significant
# GO BP term: "transport":
> head(all.GO[all.GO$GO.desc=="transport",], 1)
    aspect aspect.id         GO   GO.desc
412     BP       412 GO:0006810 transport
> head(GO.annot[GO.annot$GO=="GO:0006810",], 1)
   aspect id         GO aspect.id
54     BP 16 GO:0006810       412

# Example of the use of browse.GO()
> browse.GO("GO:0006810") # OK!
# Shown as Figure 8.

> temp.ids <- intersect(unique(GO.annot$id[GO.annot$GO=="GO:0006810"]), genes.ASm0SRm60.TF$id)
> length(temp.ids)
[1] 8
> genes.ASm0SRm60.TF.transport <- gene[is.element(gene$id, temp.ids),]
```

```
> genes.ASmOSRm60.TF.transport
         id        ProtId
3018   3018 XP_016543845.1
3286   3286 XP_016570044.1
5075   5075 XP_016555570.1
7243   7243 XP_016539755.1
18988 18988 XP_016561001.1
21751 21751 XP_016569628.1
27787 27787 XP_016547549.1
31629 31629 XP_016566495.1
                                                     Prot.Desc isTF
3018  zinc finger CCCH domain-containing protein 30-like isoform X1 TRUE
3286                                  transcription factor MYB1R1 TRUE
5075                                 bZIP transcription factor 53 TRUE
7243                               transcription factor HBI1-like TRUE
18988                             transcription factor MYB44-like TRUE
21751                                 target of Myb protein 1-like TRUE
27787                    splicing factor U2af small subunit B-like TRUE
31629                                 transcription factor bHLH104 TRUE

# Example of the use of browse.gene()
# (with the gene with id=3018, which corresponds to
# "XP_016543845.1" which in turn is described as
# "zinc finger CCCH domain-containing protein 30-like isoform X1").
> browse.gene("XP_016543845.1")
# Presented as Figure 9.
```

Box 7 illustrates the way to isolate SEPs that will contain genes that are annotated as Transcription Factors (TF) using the option '`isTF`' of function '`get.SEP()`'. In the particular case of the set '`ASmOSRm60`', we find that there are 53 TF which mean SEPs are presented in Figure 8. We can notice that the mean SEPs for the TFs in Figure 8 have the same shape than the SEPs for the whole set of 758 genes of the `ASmOSRm60` gene set, presented in Figure 7.

From **Box 7** it is also interesting to see that the proportion of TF in the set of `ASmOSRm60` genes, $\approx 7.1\%$, is larger than the percentage of TF in the whole gene set, $\approx 6.1\%$. We can also see the descriptions of the proteins coding for the 53 TFs present in the `ASmOSRm60`; **Box 7** shows only some of them.

Other interesting aspect of the TFs present in the set `ASmOSRm60` is to see which of them are annotated in particular GO terms, in particular those that resulted significantly enriched for a given biological process (BP), for example "transport". **Box 7** also shows how to isolate and see the descriptions of such genes.

Having a given list of GO terms or particular genes of interest, the researcher could want to see more general details than the ones that exist within "*Salsa*". This can be easily achieved by the use of functions '`browse.GO`' and '`browse.gene`', respectively. The use of those functions is also shown in **Box 7**, and results of the windows opened in the browser are presented in figures 9 and 10, respectively.

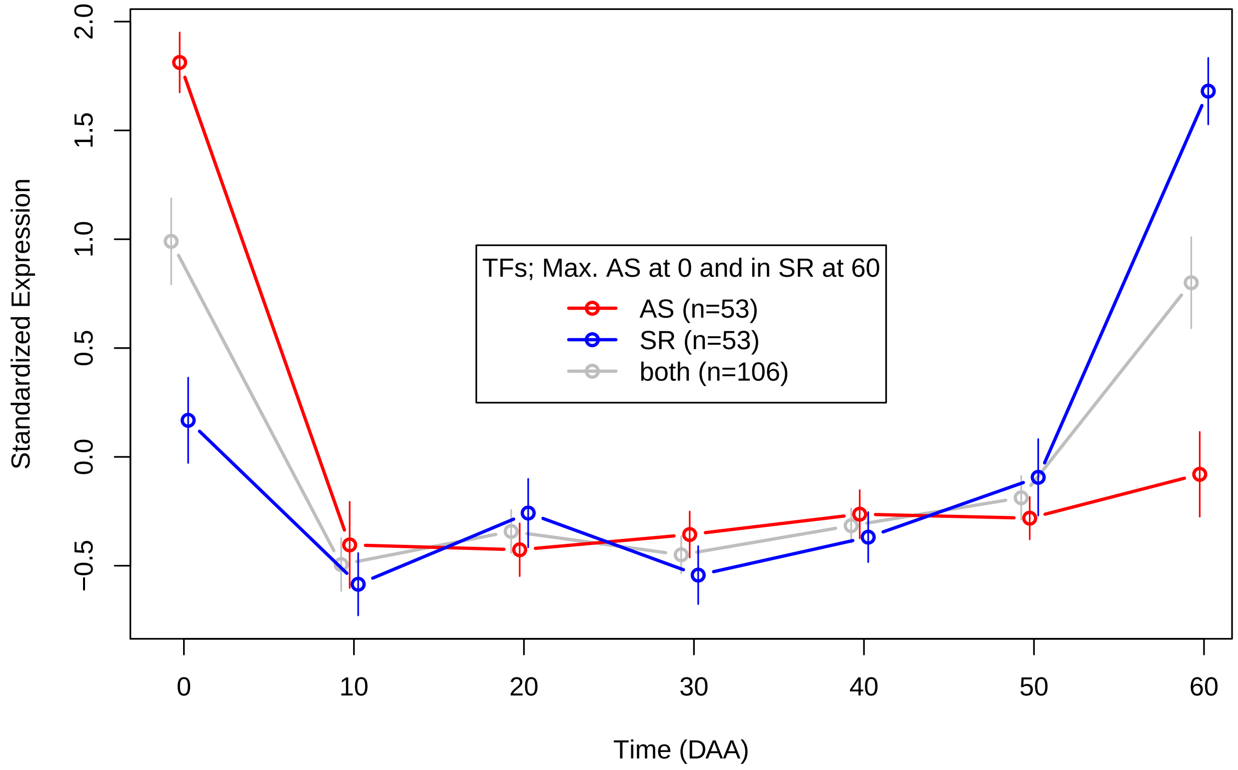

FIGURE 8. Expression profiles for Transcription Factors (TFs) simultaneously having the maximum expression at 0 DAA in accession "AS" but maximum expression at 60 DAA in accession "SR" (set "`ASm0SRm60.TF`"). Obtained with function "`SEPs.plot()`"; see **Box 7**.

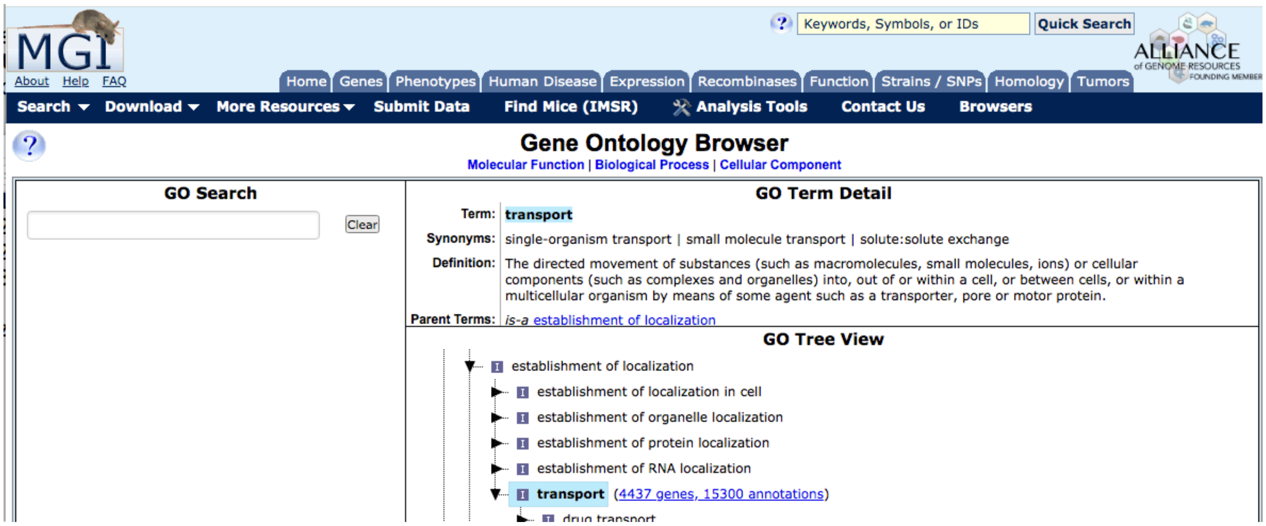

FIGURE 9. Partial view of the window open at www.informatics.jax.org. Obtained with function "`browse.GO("GO:0006810")`"; see **Box 7**.

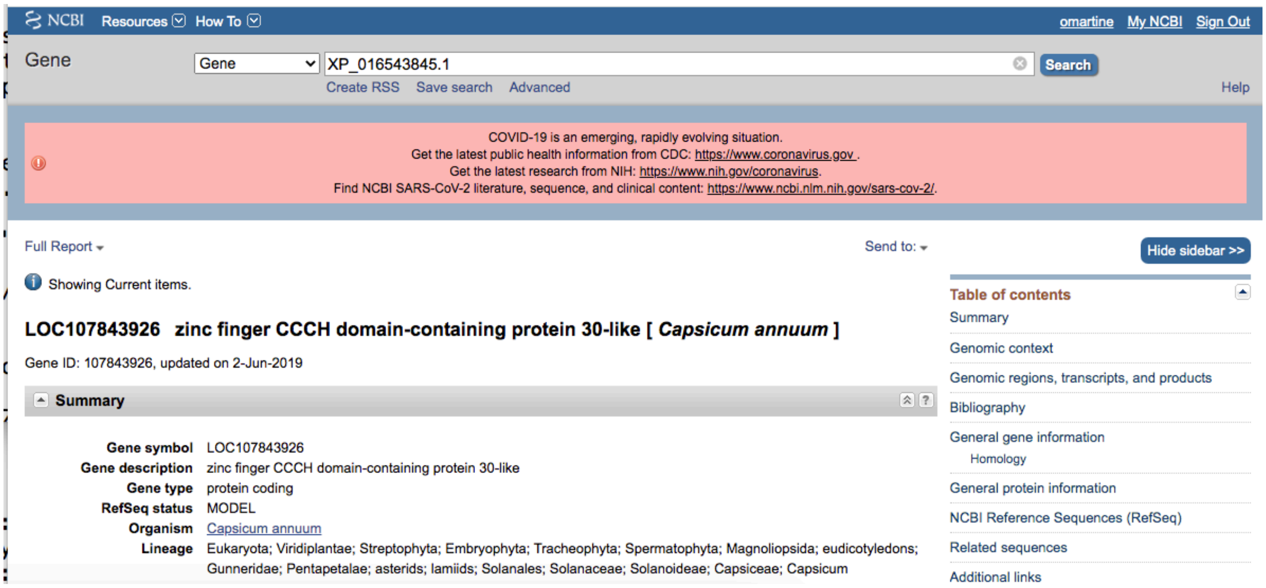

FIGURE 10. Partial view of the window open at NCBI gene. Obtained with function "`browse.gene("XP_016543845.1")`"; see **Box 7**.

3.4. **A general summary for a given gene.** For any of the 29946 genes expressed in one or more of the accessions, "*Salsa*" can give a plot and a numeric summary, extracted from the `data.frame` '`SEP.id`'. This is illustrated in **Box 8**, and the resulting plot is presented in Figure 11.

Box 8

```
-----------------------------------------------------------------------------------------
# Using gene.summary to obtain a general summary
# for gene with id=3018
> gene.summary(id=3018, leg.pos="topleft")

 id = 3018 (zinc finger CCCH domain-containing protein 30-like isoform X1)

Number of accessions per group:
Total     D     W     C
   12     6     4     2

Mean correlations per group:
 m.r.t  m.r.D  m.r.W  m.r.C
0.3833 0.3348 0.2517 0.5545

Means and Standard Deviations (S) for Maximum Expression
       Mean     S
Total 46.67 23.48
D     38.33 29.94
W     52.50 15.00
C     60.00  0.00
# Presented as Figure 10

# Try gene.summary(76) for a case where the
# expression is identical in all accessions.
-----------------------------------------------------------------------------------------
```

In **Box 8** we used the gen with identifier ('`id`') 3018, which constitutes the first row of the genes isolated in the '`genes.ASmOSRm60.TF.transport`' gene set (see **Box 7**). In **Box 8** the first line of the results give the identifier of the input and between parenthesis the description of the protein coded by the gene (column '`Prot.Desc`' in the '`gene`' data frame). The following lines in the output give the table of the number of accessions where the gene was expressed; for the case of interest ('`id=3018`'), we see that it was expressed in all 12 accessions; the 6 domesticated, ("D"), the 4 wild, ("W"), and the two crosses, ("C").

The next section in the output, entitled '`Mean correlations per group`', presents the average of the estimated Pearson correlation values ($\hat{r}$) that were estimated in the different groups of SEPs. The mean correlation between SEPs is estimated in the groups 'Total' ('`m.r.t`'), Domesticated ('`m.r.D`'), Wild ('`m.r.W`') and Crosses ('`m.r.C`'). The number of correlation coefficients averages depends on the number of SEPs present in each group, n. In all cases the number of correlation coefficients averaged is equal to $n(n-1)/2$, thus, for example, in the case illustrated in **Box 8**, we have that $n = 12$ for the total group, and thus the average was obtained from $12(12-1)/2 = 66$ individual $\hat{r}$ values, obtained for all possible 66 different pairs of SEPs, and in a similar way for the other sets of SEPs. In general, the mean correlations per group gives a very good estimation of the consistency of the expression profiles in the different groups. Large values of $\hat{r}$, say $\hat{r} \approx 1$, indicate that the profile expression in the accessions involved are highly similar (try '`gene.summary(76)`' for a case where $\hat{r} = 1$), while $\hat{r} \approx 0$ or $\hat{r} < 0$ indicate a high dissimilarity of SEPs, or, equivalently, a low expression consistency.

Finally, the last part of the function's output, entitled '`Means and Standard Deviations (S) for Maximum Expression`' evaluates the maximum peak expression of the SEPs, again in the Total, D, W and C groups. In the specific case shown in **Box 8** the large standard deviations for the means of the peak expression indicate the highly heterogeneous individual values of the SEP's peaks, which in turn are due to the 'U' shape that individual SEPs present (see Figure 8 for the average behavior of the TFs).

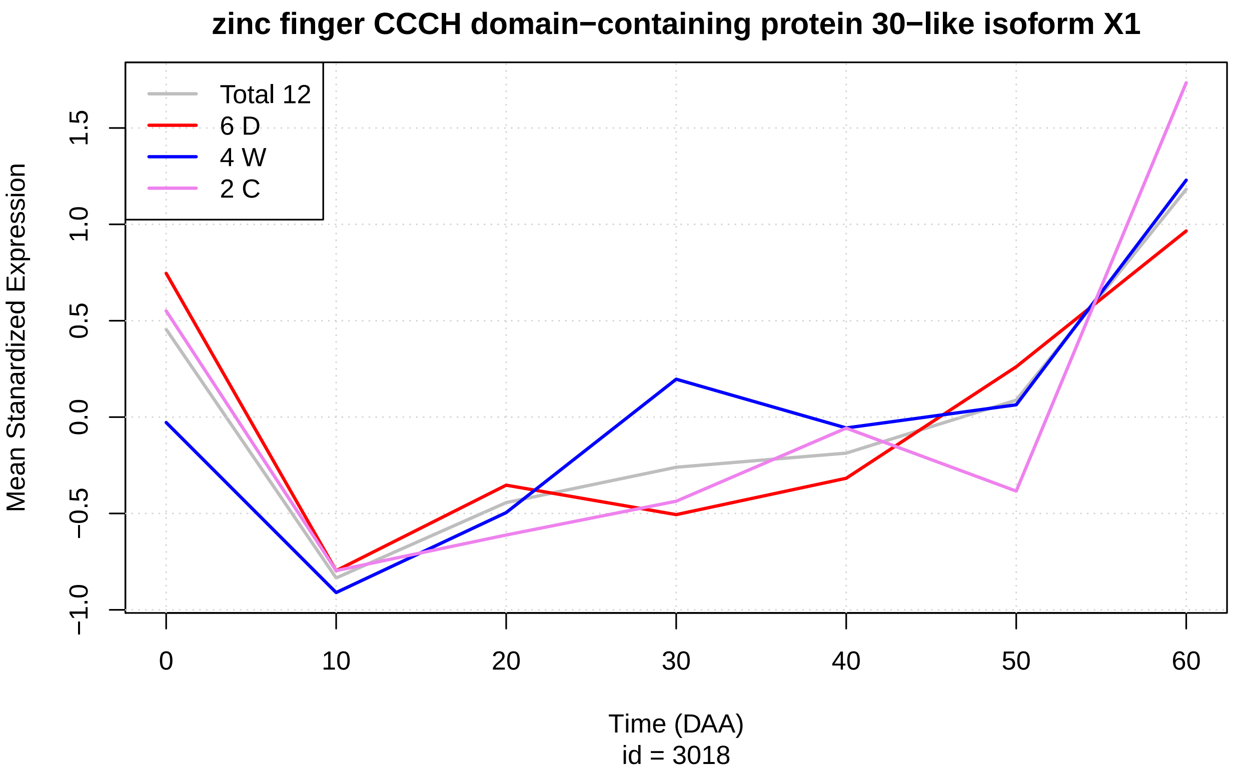

Figure 11. General Expression profiles for gene with "`id = 3018`". Obtained with function "`gene.summary(3018)`"; see **Box 7**.

In Figure 11 we see the plot produced by the function '`gene.summary(id=3018)`' (**Box 8**). From this figure we can notice that the mean standardized expression of this gene presents an approximately 'U' shaped average SEP in all groups of accessions, with a peak at 60 DAA, but significant local maxima at 0 DAA.

References

Chakrabarti S, Ester M, Fayyad U, Gehrke J, Han J, Morishita S, Piatetsky-Shapiro G, and Wang W (2006) Data mining curriculum: A proposal (version 1.0). *Intensive Working Group of ACM SIGKDD Curriculum Committee*, 140.

Edgar R, Domrachev M, and Lash AE (2002) Gene Expression Omnibus: NCBI gene expression and hybridization array data repository. *Nucleic acids research*, 30, 207–210.

Hayano-Kanashiro C, Gámez-Meza N, and Medina-Juárez LÁ (2016) Wild pepper *Capsicum annuum* L. var. *glabriusculum*: Taxonomy, Plant Morphology, Distribution, Genetic Diversity, Genome Sequencing, and Phytochemical compounds. *Crop Science*, 56, 1–11.

Martínez O, Arce-Rodríguez ML, Hernández-Godínez F, Escoto-Sandoval C, Cervantes-Hernández F, Hayano-Kanashiro C, Ordaz-Ortiz JJ, Reyes-Valdés MH, Razo-Mendivil FG, Garcés-Claver A, and Ochoa-Alejo N (2021) Transcriptome Analyses throughout Chili Pepper Fruit Development Reveal

Novel Insights into the Domestication Process. *Plants*, 10. doi:10.3390/plants10030585. URL https://www.mdpi.com/2223-7747/10/3/585.

Martínez O and Escoto-Sandoval C (2021) *Salsa: An R package of data mining facilities for Capsicum gene expression profiles*. doi:10.5281/zenodo.4767445. URL https://zenodo.org/record/4767445#.YKJmFGauJn5. This research was funded by the Consejo Nacional de Ciencia y Tecnología, México (Conacyt) project number 1570.

Spies D and Ciaudo C (2015) Dynamics in transcriptomics: advancements in rna-seq time course and downstream analysis. *Computational and structural biotechnology journal*, 13, 469–477.

Wang Z, Gerstein M, and Snyder M (2009) RNA-Seq: a revolutionary tool for transcriptomics. *Nature reviews genetics*, 10, 57–63.
